# Dataset for surface and subsurface characterization of removable urban pavements with a functionalized surface (RUP-FS) using multi-technique measurements

**DOI:** 10.1016/j.dib.2026.112921

**Published:** 2026-06-03

**Authors:** Grégory Andreoli, Margarita Skamantzari, Franziska Schmidt, Amine Ihamouten, Alexis Cothenet, Eric Gennesseaux, Nikolaos Bakalos, Thierry Sedran

**Affiliations:** a Gustave Eiffel University, MAST/EMGCU Salon-de-Provence, France; b Institute of Communications and Computer Systems, Athens, Greece; c Gustave Eiffel University, MAST/EMGCU Marne-la-Vallée, France; d Gustave Eiffel University, MAST/LAMES Nantes, France; e Gustave Eiffel University, MAST/MIT Nantes, France

**Keywords:** RUP, Imagery, 3D GPR, YOLO

## Abstract

The evolution of transportation infrastructure has become an increasingly critical issue as extreme weather events intensify and traffic density grows, leading to a rise in visible surface degradation. In addition, mechanical stresses on pavements and potential infrastructure settlements have significant impacts on underground utility network nodes [1]. Regardless of the type of degradation, innovative solutions are required to design the roads of the future. In this context, an innovative system called Removable Urban Pavements (RUP) has been developed. Its purpose is to make easier and faster the access to the base layer and underground utility networks during maintenance operations. This modular structure can be opened and closed quickly, thereby reducing intervention time and preventing the longitudinal unevenness typically observed after trenching on standard pavements. The RUP structure consists of interlocking, prefabricated hexagonal slabs made of porous concrete with a draining surface [2], which helps prevent standing water and reduces the risk of hydroplaning.

To characterize the condition of RUP and ensure reliable monitoring of their evolution, Non-Destructive Testing (NDT) methodology combining imaging and electromagnetic (EM) wave propagation has been implemented. Structural identification is performed using a drone (Unmanned Aerial Vehicle: UAV), enabling element-by-element detection and classification between RUP and standard pavements. Surface anomalies are detected from ground-level images captured with a smartphone acting as a stand-in for a robot (Unmanned Ground Vehicle: UGV) developed within the European HERON project [3]. Subsurface condition visualization is achieved using a Stepped-Frequency 3D Ground Penetrating Radar (GPR) system [4]. The ultimate goal is to develop an evolving database containing both structural components and the various detectable anomalies. A deep learning model based on a YOLO-type (You Only Look Once) architecture can then be used to perform automatic classification of these elements.

Specifications Table

**Table:** 

Subject	Engineering & Materials science
Specific subject area	Removable Urban Pavement Characterization methods.
Type of data	Pictures (*.jpg); Data base YOLO format (*.txt); 3D GPR (*.3dra, *.3dp): open with Examiner 3.5.0 and a conversion in seg-Y format for each onel
Data collection	This database is created using UAV DJI 3S device, smartphone Samsung S21 Ultra with HD camera and Stepped-Frequency 3D GPR with DXG1820 antenna array from KONTŪR on Removable Urban Pavement structures. All the surface data are collected and labeled with the open source on line software Make Sense and export in a text YOLO format.
Data source location	Experimental dataset on the fatigue carousel of Gustave Eiffel University (Nantes Campus) Location: 47°09′13.0"N 1°38′30.0"Wl
Data accessibility	Repository name: Dataverse: recherche.data.gouv.fr Data identification number: 10.57745/37WIXB Direct URL to data: 10.57745/37WIXB
Related research article	• The dataset is relative to the article DOI: Revision in progress (pending DOI) • Named: Experimental Multi-Technique Characterization of Structural Anomalies in Removable Urban Pavements with Functionalized Surface (RUP-FS)

## 1 Value of the Data

• The datasets consist of collections of images acquired using an UAV at different altitudes to enable the identification of the Removable Urban Pavements (RUP) structure, as well as ground-level images captured with a smartphone to support future classification of various surface anomalies (scaling, staining, etc.). The datasets also include 3D GPR data revealing the subsurface structural condition through A-scans, B-scans, cross-scans, and C-scans;
• The RUP structure is a full-scale pavement system with controlled material formulation and geometry produced in a laboratory environment. Its uniform geometry across the entire test area enables precise comparisons of the structural condition from one slab to another, making it possible to identify local singularities;
• The generated databases compile the slab types and defect types, which are delimited and labelled using anchor boxes suitable for deep-learning algorithms as YOLO architectures;
• The 3D GPR files support the understanding of wave/material interactions in complex geometric structures such as the RUP;
• These experimental datasets, acquired using different measurement techniques, are shared with the broader scientific (university) and professional pavement community, particularly those working with imaging and Stepped-Frequency 3D Ground Penetrating Radar (GPR). Road and highway operating companies are therefore the primary stakeholders that can ultimately benefit from this type of data. They are intended to support the detection and characterization of emerging innovative pavement structures in the context of the Forever Open Road (FOR) [5] initiative and its core principles: resilience, automation, and adaptability, developed by the Forum of European National Highway Research Laboratories (FEHRL).

## 2 Background

Based on an innovative concept proposed by Gustave Eiffel University in 2008, Removable Urban Pavements (RUP) were designed to provide rapid and easy access to the base layer and underground network for potential maintenance needs. This modular structure can be opened and closed using a system of prefabricated hexagonal concrete slabs that interlock with one another. A concrete key integrated at the base of each slab during manufacturing limits the risk of tilting and localized settlement under heavy traffic.

**Fig. 1:**
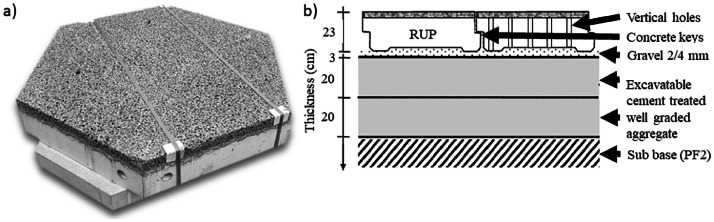
a) Hexagonal RUP-FS; b) RUP-FS implementation on a structure [4].

Within the I-Street project, initiated in September 2017, RUP systems evolved into permeable RUP with a functionalized surface (RUP-FS) (Fig. 1a) [6]. This new feature improves resilience to increasingly extreme weather events. Vertical void tube located beneath the porous concrete surface, serving as the wearing course, allow water drainage, reducing the risk of surface hydroplaning. The RUP structure is built on a cement-treated, excavatable, and permeable base layer (Fig. 1b).

To test various pavement structures, the Nantes campus of Gustave Eiffel University (France) has been operating a unique full-scale testing facility for over 40 years known as the “fatigue carousel” [7,8]. Its principle is to accelerate pavement aging using a rotating system of four weighted arms that simulate heavy-vehicle loading, reaching speeds of up to 90 km/h. In this study, an RUP-FS structure was subjected to 200,000 loading cycles over two months, corresponding to regular bus traffic (between 45 and 91 passes per day in both directions) over a 20 years’ period [9].

The datasets collected in this study include drone (UAV) imagery at multiple altitudes and high-definition smartphone images, simulating an unmanned ground vehicle (UGV) developed within the European HERON project (automated road inspection and repair) [10,11]. Finally, subsurface datasets were acquired using an electromagnetic (EM) wave-propagation device to assess both intrinsic and extrinsic subsurface structural conditions.

## 3 Data Description

The database consists of drone (UAV) and smartphone (UGV) pictures of the surface of the RUP-FS structure, along with 3D GPR data characterizing the subsurface condition. The data structure tree is described as follows:

•  Experimental_RUP-FS_Database
•  /Fatigue_Carousel_Experimental_area
  •  /UAV
    •  /Data_UAV_Carousel_Pictures
      •  DJI_xxxx.jpg
    •  /Data_UAV_Carousel_YOLO_TXT
      •  DJI_xxxx.txt
    •  Data_UAV_Carousel.txt
    •  Data_UAV_Carousel.csv
  •  /UGV
    •  /Data_UGV_Carousel_Pictures
      •  RUP_xxxx.jpg
    •  /Data_UGV_Carousel_YOLO_TXT
      •  RUP_xxxx.txt
    •  Data_UGV_Carousel.txt
    •  Data_UGV_Carousel.csv
  •  /3DGPR
    •  /TW25ns
      •  FileOuput
        •  *.3drnav
        •  *.3drvol
        •  *.3dra
      •  RegionOutput
        •  *.3drvol
      •  2025-01-17-004.3dra
      •  2025-01-17-004.segy
      •  2025-01-17-005.3dra
      •  2025-01-17-005.segy
      •  2025-01-17-006.3dra
      •  2025-01-17-006.segy
      •  TW_25ns.3dp
      •  TW_25ns.segy
    •  /TW35ns
      •  FileOuput
        •  *.3drnav
        •  *.3drvol
        •  *.3dra
      •  RegionOutput
        •  *.3drvol
      •  RegionOutput
      •  2025-01-17-007.3dra
      •  2025-01-17-007.segy
      •  2025-01-17-008.3dra
      •  2025-01-17-008.segy
      •  2025-01-17-009.3dra
      •  2025-01-17-009.segy
      •  TW_35ns.3dp
      •  TW_35ns.segy

At the first level, the directory corresponds to the test area. The second level specifies the equipment used (UAV, UGV, 3DGPR). At the third level, for imaging data, find folders containing the raw photos (.jpg format) and annotation files following the YOLO anchor-box format (.txt). For 3D GPR data, subfolders are organized according to the time window duration used (/TW25ns or /TW35ns). 3D GPR data are provided in the .3dra format for geolocated raw acquisition files and .3dp for stitched files (combining multiple 3D GPR passes into a single panoramic image), with and without Kirchhoff migration. It is possible to read it with the software EXAMINER v3.5.0 (KONTÜR). In addition, the *.3dra and *.3dp files have also been converted into the SEG-Y (*.segy) format, allowing broader interoperability and more universal accessibility with standard geophysical and signal-processing software.

The FileOutput and RegionOutput directories contain files with the extensions *.3drnav, *.3drvol, and .3dra, which are specific to the history of optimal parametric configuration settings used for 3D GPR data reading (filtering, processing, etc.). These files cannot be opened directly. To modify these settings, an EXAMINER 3.5.0 license is required, by opening the .3drp and .3dra files.

Each photo taken with UAV represents part of the structure (or the full structure depending on altitude) and is numbered chronologically. Each one is linked to a .txt file containing the class number and the anchor-box coordinates (see table below).

UAV photos follow the naming convention: 



Two additional files named "Data_UAV_Carousel.txt" and "Data_UAV_Carousel.csv" compiles all of the individual UAV .txt files.

Each photo taken with smartphone (UGV) represents at least one slab named according to its position in the structure (see Fig. 3b) :

Smartphone (UGV) photographs follow the convention:  and



Two additional files named "Data_UGV_Carousel.txt" and "Data_UGV_Carousel.csv" compiles all of the individual UAV .txt files.

For the 3D GPR device, 3 acquisition files (*.3dra) represent 3 passes on the structure for each listening window (25 and 35 ns). Additionally, one .3dp file groups the 3 files for each window, totaling 8 files.

3D GPR acquisition files follow the format: 



Stitched 3D GPR files follow:



The RUP structure is an emerging one. This directory structure is designed to be flexible and scalable. Additional folders can be created based on the test area, the equipment used, the data type, or the time window (for 3D GPR).

## 4 Experimental Design, Materials and Methods

**Fig. 2:**
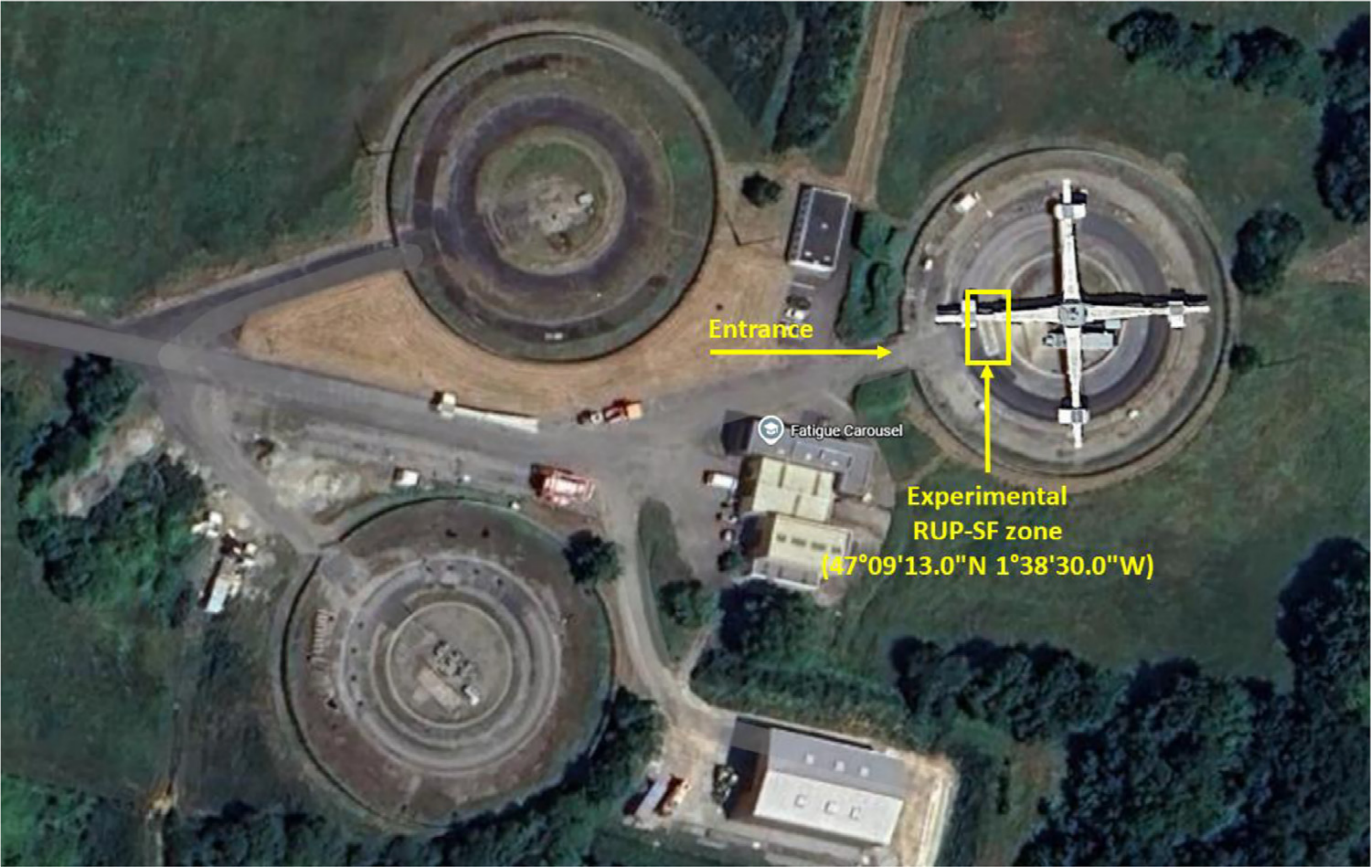
Fatigue carousel at Gustave Eiffel university in Nantes (France).

The fatigue carousel (Fig. 2) at Gustave Eiffel University in Nantes is a full-scale pavement testing facility engineered to speed up surface and structural deterioration. The system uses four radially arranged loading arms capable of applying high wheel loads, up to 65 kN per wheel, in either tandem or tridem axle configurations. The rotation speed is variable and can be set to simulate traffic speeds of up to 90 km/h. In this study, the RUP-FS structure was exposed to 200,000 load cycles over a two-month period, representing the equivalent of routine bus traffic, approximately 45 to 91 passes per day in each direction, accumulated over a 20-year service life.

**Fig. 3:**
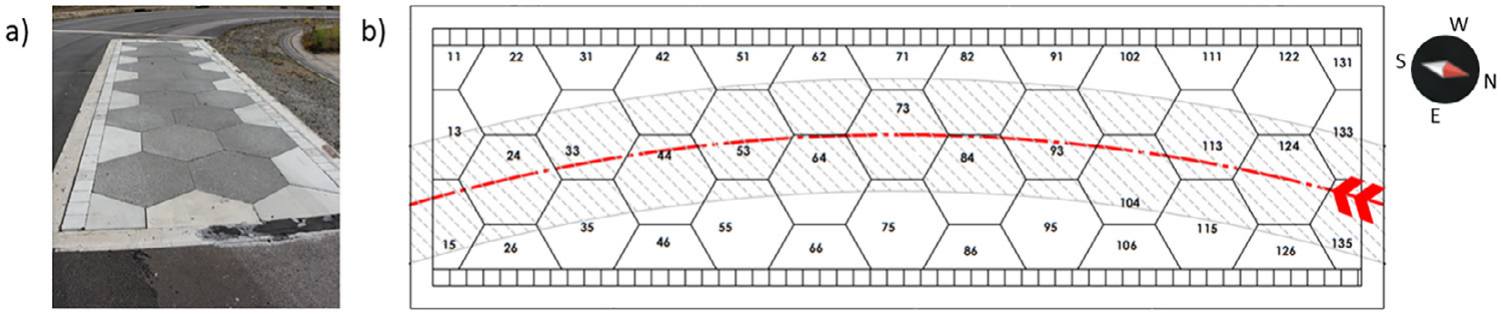
BINARY Structure on the fatigue carousel: a) Various tri-layers structure; b) Section on the carousel with direction of rotation (arrow in red) and numbering of each slab [4,9].

As part of the I-Street project, a Removable Urban Pavement with a Functionalized Surface (RUP-FS) system was deployed on the fatigue carousel to assess both its mechanical behavior and functional performance. The test section (Fig. 3a) measures 8.35×2.31 m and consists of 39 slabs, each 23 cm thick with a 46 cm side dimension. The configuration includes 15 half-slabs and 2 quarter-slabs fabricated from hydraulic concrete, while the remaining 22 hexagonal slabs incorporate a 19 cm hydraulic concrete base topped with a 4 cm porous concrete layer designed to enhance drainage. In addition, 108 squares concrete cobblestones (and 2 half one on the corners) were inserted longitudinally to allow for dismounting of the structure. The experimental objective was to characterize the slabs’ mechanical performance, acoustic behavior, and drainage capability.

**Fig. 4:**
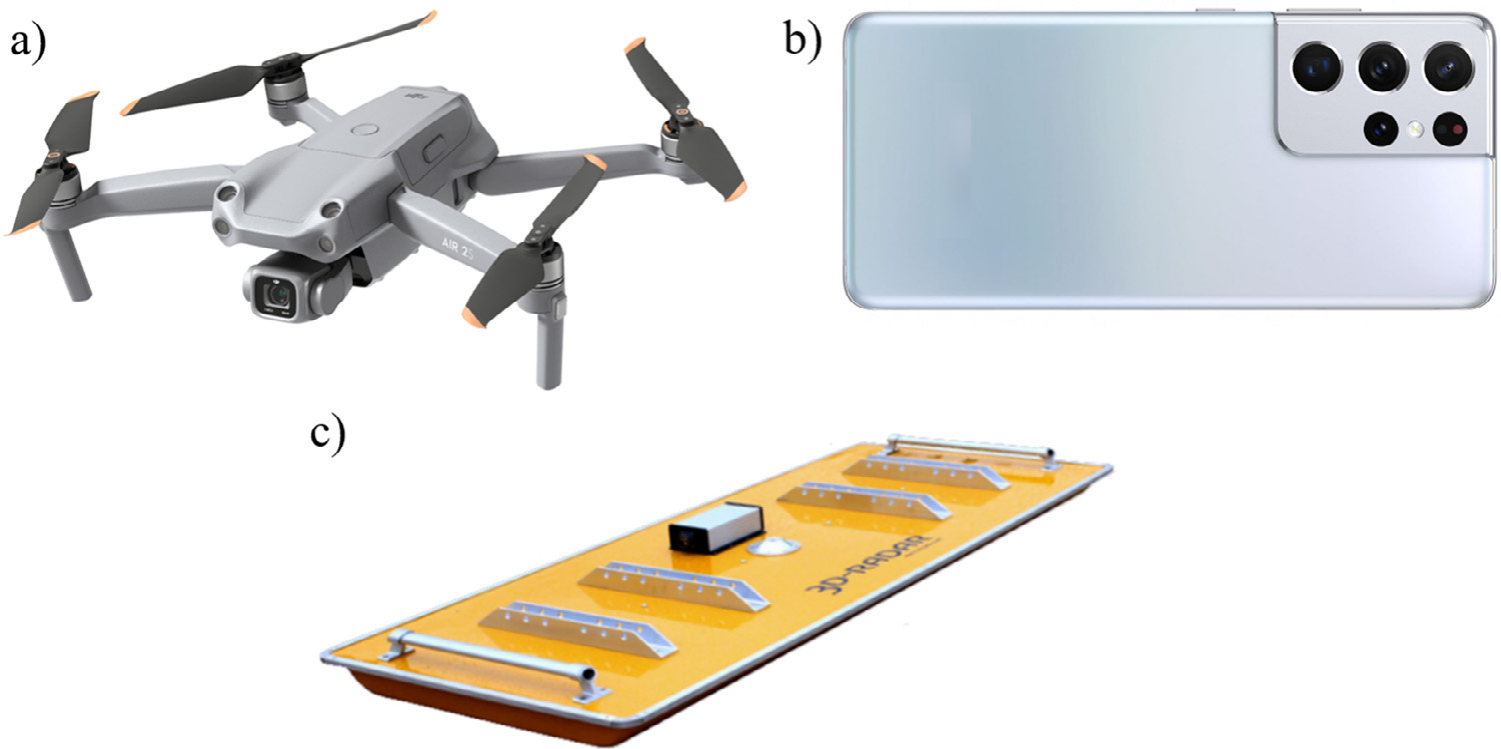
a) UAV DJI 3S; b) Smartphone; c) Ground coupled antennas 3D GPR (KONTÜR) [4].

It is important to note that all data were collected under weather conditions suitable for surface imaging—limited cast shadows, consistent sunlight, and dry pavement. No rainfall occurred during the week preceding the measurements, minimizing the presence of moisture within the top 4 cm of the porous concrete slabs, which is critical for the 3D GPR surveys. The extraction of quantitative information about the controlled structure is allows using UAV DJI 3S (Fig. 4a), a Smartphone Samsung S21 Ultra (Fig. 4b) and a 3D GPR multichannel antenna network DXG1820 with Stepped-Frequency 3D GPR (manufactured by KONTÜR) (Fig. 4c).

**Fig. 5:**
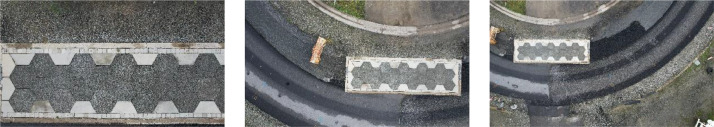
Example of RUP-FS UAV pictures: a) 5m in altitude; b) 10m in altitude; c) 15m in altitude.

**Fig. 6:**
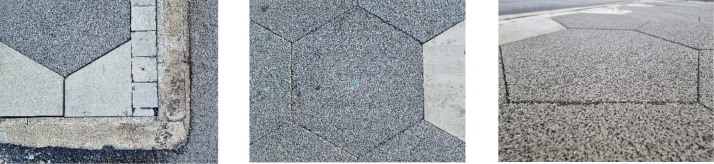
Example of RUP-FS smartphone pictures: a) Quarter slab; b) Hexagonal and half slab; c) Various angle view.

The methodology and weather for each device is:

1) Drone (UAV) (Fig. 5):

• Model: DJI 3S,
• Photo dimensions: 5472×3078 pixels,
• Format: jpg,
• Number of photos taken on the fatigue carousel: 118,
• Altitude: 5 m, 10 m, 15 m (and some random intermediate altitude),
• Date: 2024-02-22

2) Smartphones (UGV) (Fig. 6):

• Model: Samsung S21 Ultra and A56,
• Photo dimensions: 3000×4000 pixels,
• Format: jpg,
• Number of photos taken: 59 (39 top views and 20 oblique views to observe vertical differences between slabs),
• Height for top views: 1 meter (allowing full visualization of a hexagonal slab with sufficient overlap),
• Date: 2023-11-22 and 2023-11-24.

For the UGV surrogate acquisition, the smartphone was manually held at approximately 1 m above ground level. This setup was intentionally chosen to preserve a low-height ground perspective while introducing minor pose and overlap variations, thereby capturing a more diverse and realistic set of viewpoints. The surrogate should therefore be interpreted as a practical proxy for ground-level acquisition rather than a rigidly stabilized UGV mount.

**Fig. 7:**
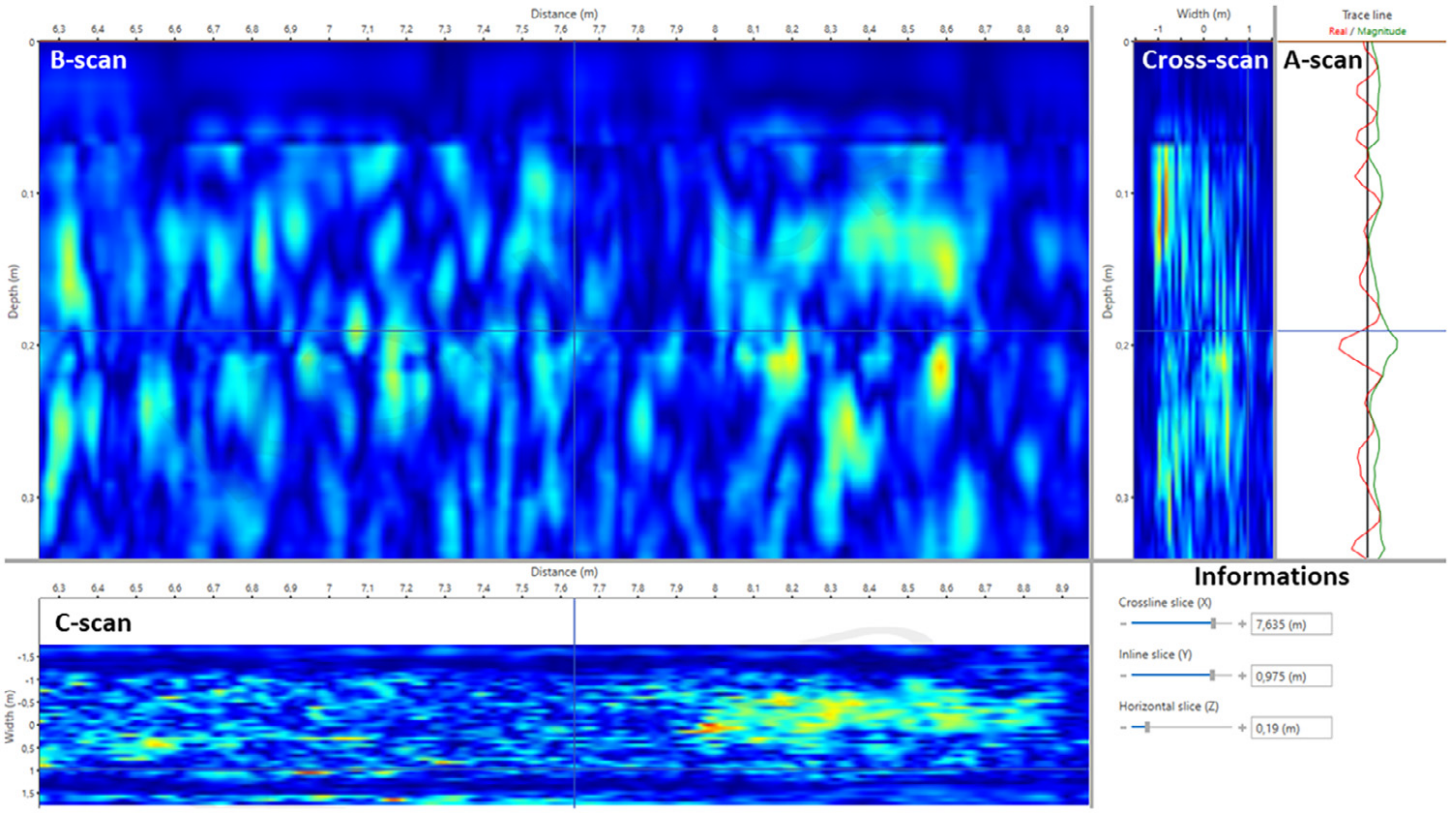
Example of RUP-FS subsurface structure 3D GPR signal opened with EXAMINER 3.5.0 software.

3) Stepped-Frequency 3D GPR with DXG1820 antenna array from KONTÜR (Fig. 7):

• Frequency band = [0.2 – 2.98 GHz],
• Listening window: 25 ns and 35 ns,
• Measurement step: 1 cm,
• Format: 3dra, 3dp (proprietary format readable with EXAMINER 3.5.0 from KONTÜR),
• Height: ground-coupled,
• Date: 2025-01-23 (11:40 AM to 11:52 AM).

To ensure sufficient overlap in the 3D GPR data, three passes were performed for each time window, following the same direction as the carousel’s rotation:

• **2025-01-17-004.3dra** and **2025-01-17-007.3dra** (toward the carousel center),
• **2025-01-17-005.3dra** and **2025-01-17-008.3dra** (over the center of the RUP-FS structure),
• **2025-01-17-006.3dra** and **2025-01-17-009.3dra** (toward the outer edge of the carousel).

Based on the multi-technique data collected, it is possible to identify the RUP-FS structure (with UAV) and surface anomalies (with UGV), as well as to observe the differences between healthy and deteriorated conditions in the subsurface (3D GPR).

The objective is to have a representative and evolving database of RUP-FS enabling their identification and experimental characterization. The UAV must be able to distinguish the different elements of the structure, the smartphone simulating a UGV must be able to identify surface anomalies of the structure and finally the 3D GPR must be able to differentiate healthy slabs from structural anomalies.

The raw surface datasets collected can be considered incomplete due to their limited representativeness (absence of shadow projections, localized moisture spots, and urban pollution-related artifacts), as well as the relatively small number of training images (118 aerial images and 59 ground-based images). Consequently, data augmentation is recommended to artificially increase dataset variability. The augmentation process can generate additional samples through geometric transformations (such as rotation, flipping, and scaling) and variations in the HSV color space (Hue, Saturation, Value). These preprocessing steps increase the number of training samples, thereby improving model performance while ensuring a more balanced dataset size [4].

Surface data were labeled using anchor boxes (UGV: Table 1; UAV: Table 2) with the free *Make Sense* software [13] and annotated according to the following nomenclature:

**Table 1:** Annotation for Smartphone (UGV) photos.

**Table 2:** Annotation for UAV photos.

The .txt file represents the entire database (without header) for each recording device (drone or smartphone) and contains:

**Table:** 

Column 1	Column 2	Column 3	Column 4	Column 5	Column 6
Filename	class number	YOLO Anchor-box format			
*.jpg	[0 – 6]	x center	y center	width	height

**Fig. 8:**
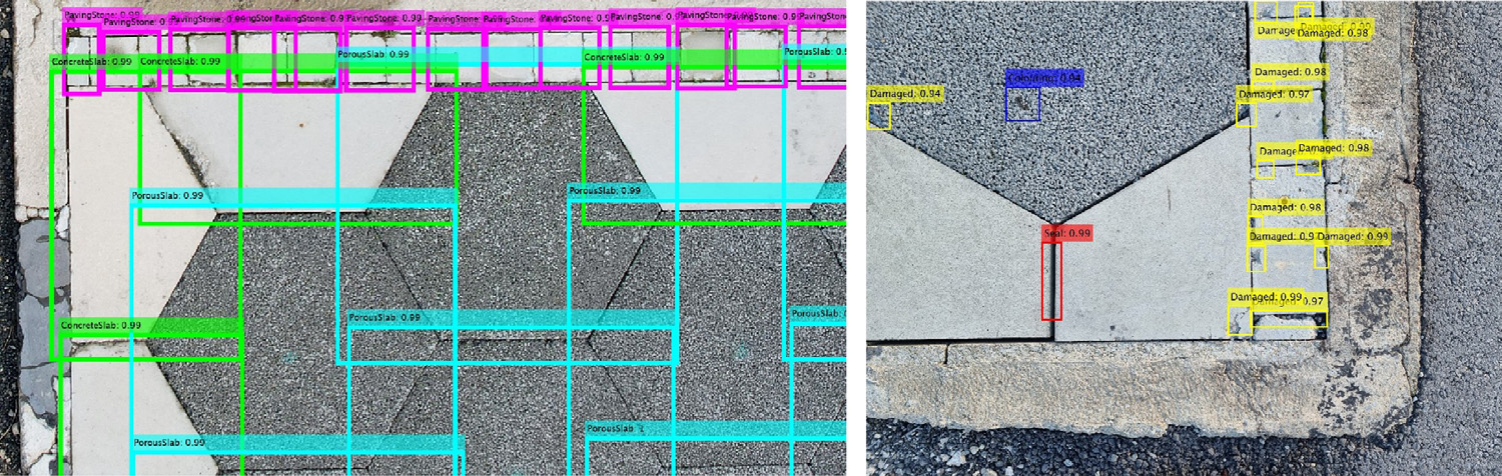
Example of Anchor-box: a) RUP-FS identification with UAV: b) RUP-FS anomalies classification (smartphone/UGV).

Thanks to this evolving database, deep learning, based algorithmic models, such as those built upon a YOLO architecture, can be deployed to support the detection and classification of structural components (UAV) as well as surface anomalies (UGV). A multiclass detection framework such as YOLOv4 is particularly well suited to this task (Fig. 8) [4].

## Limitations

• This study relies on a limited but structured dataset composed of 118 UAV images and 59 smartphone images. As a result, the present analysis should be interpreted as an initial validation of the proposed pipeline rather than as a fully exhaustive assessment of field variability. Data augmentation and further data collection are therefore considered necessary to improve generalization. For the UAV data, the core of the model relies on identifying the distinctive geometric pattern of the hexagonal RUP-FS slabs, discriminating their macrotexture from half/quarter slabs, and detecting the boundary cobblestones surrounding the structure. The RUP-FS components are prefabricated, ensuring controlled and highly repeatable production conditions, which are well suited for YOLO-type detection and classification algorithms.
• All acquisitions were conducted under clear and dry environmental conditions, with limited cast shadows and relatively stable illumination. Although this configuration was required for the corresponding 3D GPR measurements, it can introduce a contextual bias for the picture’s dataset (UAV and Smartphone). Consequently, the proposed YOLO-based approach should be interpreted with caution when transferred to inspection scenarios involving moisture, glare, heterogeneous shading, or more variable outdoor lighting conditions. This limitation should be considered when reusing the dataset for future machine learning studies.

## Ethics Statement

The present work did not involve the use of human subjects, animal experiments, or data collected from social media platforms.

## CRediT Author Statement

**Grégory Andreoli:** Conceptualization, Methodology, Investigation, smartphone and 3D GPR acquisition, write original draft; **Margarita Skamantzari:** UAV Acquisition; **Franziska Schmidt:** Conceptualization, Supervision, Writing and Review & Editing, Validation; **Amine Ihamouten:** 3D GPR acquisition, Writing and Review & Editing, Validation; **Alexis Cothenet:** 3D GPR acquisition; **Eric Gennesseaux:** Conceptualization, Investigation; **Nikolaos Bakalos:** Supervision; **Thierry Sedran:** Resources, Conceptualization, Investigation, Supervision.

## Data Availability

DataverseMulti-technical datasets for the evaluation of Removable Urban Pavements with Functionalized Surface (RUP-FS), assessed at the surface using UAV and UGV imaging and in the subsurface using SF-3DGPR (Reference data).

## Appendix Supplementary materials

Image, application 1
