# Supplementary material for: Dataset for surface and subsurface characterization of removable urban pavements with a functionalized surface (RUP-FS) using multi-technique measurements
Source: Data Brief. 2026 Jun 3;67:112921. doi: 10.1016/j.dib.2026.112921 (PMC13272573; doi:10.1016/j.dib.2026.112921)
Supplement: Supplementary file 1 [file mmc1.pdf]

Experimental Multi-Technique Characterization of Structural Anomalies in Removable Urban Pavements with Functionalized Surface (RUP-FS)

Grégory Andreoli, Franziska Schmidt, Amine Ihamouten, Alexis Cothenet, Eric Gennesseaux and Thierry Sedran

***Abstract*—Removable Urban Pavement with Functionalized Surface (RUP-FS), developed by Gustave Eiffel University, is an innovative modular pavement system designed to improve the resilience and adaptability of urban road infrastructure. Within the framework of the European HERON project, the research focused on the development of automated defect detection and classification methods for RUP-FS structures through a combined multi-technique and multi-physics approach. Surface condition monitoring is addressed using a deep learning algorithm based on the YOLOv4-tiny (You Only Look Once version 4 tiny) architecture applied to collected images for surface anomaly characterization. Unmanned Aerial Vehicle (UAV)-based image acquisitions demonstrated reliable detection and classification of RUP-FS components into three classes (hexagonal (full) slabs, half/quarter slabs, and concrete cobblestones), while Unmanned Ground Vehicle (UGV)-based imaging enabled accurate identification of four major surface defects (unevenness, damage, pollution-induced discoloration, and sealing defects). To complement this work, subsurface characterization is performed using Stepped-Frequency 3D Ground Penetrating Radar (3D GPR), enabling the distinction between healthy slabs (with or without metallic fibers incorporated during manufacturing) and defective slabs, including the identification of intrinsic or extrinsic anomalies. The integration of UAV and UGV-based imaging with Stepped-Frequency 3D GPR offers a methodology for Structural Health Monitoring (SHM) of RUP-FS structures, supporting predictive maintenance strategies and aligning with the Forever Open Road (FOR) vision.**

***Index Terms*—Removable Urban Pavement, Deep Learning, Imagery, Stepped-Frequency 3D GPR**

I. Introduction

The maintenance of asphalt pavements is a critical challenge for infrastructure managers due to the high costs and duration of interventions, which also impact road users [1]. Recurring maintenance work often requires repeated opening and closing of the pavement structure to access underground utility networks. Despite precautions taken during compaction phases, these interventions frequently lead to deformations, as well as issues related to waterproofing and surface uniformity, ultimately affecting user comfort and reducing the overall durability of the infrastructure.

To address these challenges, the *Laboratoire des Ponts et Chaussées* (LCPC) (Currently included in Gustave Eiffel University) and its partners developed an innovative concept for Removable Urban Pavement (RUP) between 2003 and 2008 [2, 3, 4]. These structures allow for rapid opening and closing, facilitating access to base layers and underground networks for maintenance or expansion. Following this research work, from 2017 to 2022, within the framework of the I-Street project [5], Gustave Eiffel University, in collaboration with Eiffage Route, Alkern, and *Nantes Métropole*, developed and evaluate in lab and full-scale conditions Removable Urban Pavements with Functionalized Surface (RUP-FS) (Fig. 1a) [6].

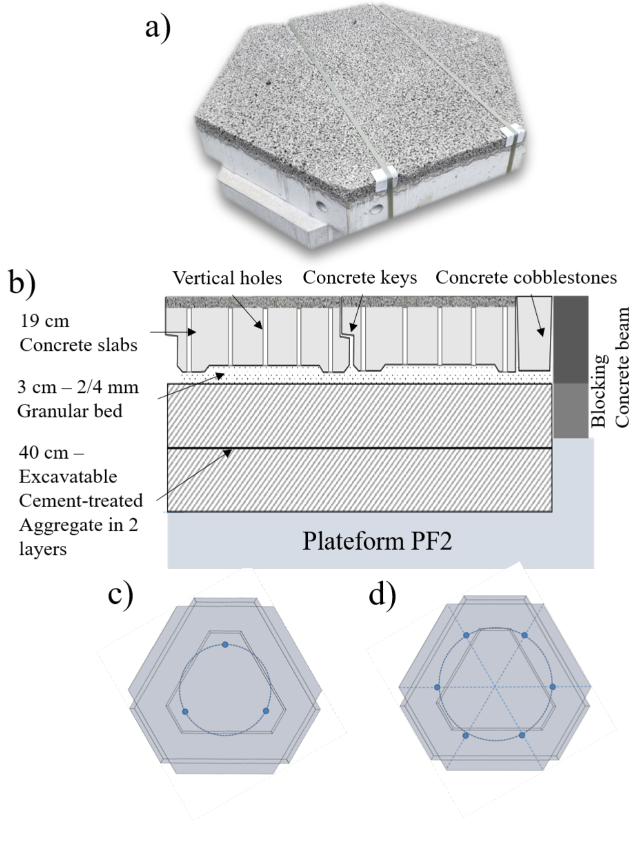

Fig. 1. a) RUP-FS (Hexagonal (full) slab); b) RUP-FS implementation on a structure; c) Slab (bottom view) with three 2.5 cm diameter holes evenly distributed along a circle with a 21.25 cm diameter; d) Slab (bottom view) with six 2.5 cm diameter holes evenly distributed along a circle with a 56 cm diameter.

G. Andreoli is affiliated with the MAST/EMGCU Research Group, Gustave Eiffel University, Salon-de-Provence, France

F. Schmidt is affiliated with the MAST/EMGCU, Research Group, Gustave Eiffel University, Marne-la-Vallée, France

A. Ihamouten is affiliated with the MAST/LAMES Research Group, Gustave Eiffel University, Nantes, France

A. Cothenet is affiliated with the MAST/LAMES Research Group, Gustave Eiffel University, Nantes, France

E. Gennesseaux is affiliated with the MAST/MIT Research Group, Gustave Eiffel University, Nantes, France

T. Sedran is affiliated with the MAST/MIT Research Group, Gustave Eiffel University, Nantes, France

RUP-FS structure is composed of interlocking precast hexagonal slabs made of plain concrete, covered with a 3 to 4 cm-thick porous concrete wearing course [7], and placed over an excavatable, traffic-resistant sublayer (Fig. 1b) [8, 9, 10, 11]. The advantage of prefabricating each slab is that it enables a controlled, monitored, and repeatable production process. Concrete interlocking keys prevent noisy slab rocking under heavy traffic [12], while vertical holes through the slabs provide efficient drainage, reducing flood risks and enhancing user safety, particularly in the context of extreme weather events. During the experimental phase of the I-Street project, slabs with three and six vertical holes were designed and tested (Fig. 1c and 1d).

The RUP-FS structure is an innovative concept, and the I-Street project [5] enabled the construction of two pilot sites. The first is located on the fatigue carousel [13] at Gustave Eiffel University (Nantes campus/France). This facility provides a controlled testing environment dedicated to accelerated pavement aging. The second site is on Allier Street, located in downtown Nantes (France), and serves as a full-scale demonstrator open to traffic. These two complementary sites offer the advantage of enabling feasibility studies on an emerging pavement structure while supporting the development of methodologies for detecting and analyzing defects that differ from those observed in conventional pavements.

However, during construction or over the service life of the RUP-FS structure, several distress types may manifest, including leveling between adjacent slabs (unevenness), edge spalling (damage), surface contamination as hydrocarbon residues or adhered foreign materials (pollution colouring), and joint opening or progressive joint widening (seal). Currently, visual inspection remains the primary method of monitoring, yet it has limitations in terms of completeness and anomaly detection. Moreover, identifying potential structural defects below the surface is impossible without partially dismantling the pavement. To enhance defect identification while ensuring operator safety, an innovative multi-technique data collection and processing methodology is required [14, 15, 16].

The European HERON project [17, 18] aligns with this objective by developing a robotic system integrating Unmanned Ground Vehicle (UGV) and Unmanned Aerial Vehicle (UAV) for predictive, preventive, and corrective maintenance [19, 20, 21, 22]. The project is framed within the Forever Open Road (FOR) concept established by the Forum of European National Highway Research Laboratories (FEHRL). In France, this concept is adapted under the term *Route de 5ème génération* (meaning 5th-generation road) [23]. The objective is, as a first step, to detect, classify and geolocate the 3 constituent elements of a RUP-FS structure using UAV, namely the hexagonal (full) slabs, the half/quarter slabs located along the perimeter, and the surrounding concrete cobblestone that frame the structure. Once these elements are clearly identified and geolocated by UAV, an intervention order is sent to UGV with the geolocation of the RUP-FS structure. UGV acquires ground-level imagery of each component to detect potential anomalies among the four previously defined (unevenness, damage, colouring by pollution or joint seal defects) [24]. These systems collect high-resolution RGB images [25] first from aerial views enabling automatic detection and classification of RUP-FS structural components and then images from ground views to detect and classify surface anomalies. To achieve this, we conducted extensive data collection on two test sites in Nantes city (France). The proposed approach is intended as a condition-monitoring component for defect detection that can feed into a broader predictive maintenance framework, rather than as a complete maintenance forecasting system. It contributes to predictive maintenance within the HERON project by enabling the detection of anomalies; however, its scope does not include failure prediction, temporal forecasting, or remaining useful life estimation.

Road infrastructure monitoring systems are subject to strong operational constraints, including real-time processing, integration on mobile platforms, and full-scale data acquisition under varying environmental conditions [26]. In this context, we propose a data processing methodology based on images captured by UAV and UGV for classification with a lightweight convolutional architecture such as YOLOv4-tiny (You Only Look Once version 4 tiny) [27]. The model provides a favorable trade-off between detection performance, inference speed, and computational efficiency [28, 29, 30]. This work focuses on developing a feasibility study for an operational and scalable anomaly detection pipeline for RUP-FS inspection, rather than on benchmarking state-of-the-art deep learning architectures. The proposed approach prioritizes generalizability and scalability over marginal accuracy gains, which is a key requirement for network-level road condition monitoring.

Additionally, to ensure the long-term integrity of the pavement, it is crucial to assess the condition of the underlying excavatable base layers. Since this information cannot be obtained through simple visual inspection, multi-channel microwave electromagnetic devices present a promising alternative using Stepped-Frequency 3D GPR to visually discriminate subsurface structural conditions. The objective is to assess the subsurface structure and continuously acquire the largest possible dataset to identify significant dielectric contrasts [31]. Moreover, given the recent construction of the RUP-FS structures, subsurface distresses remain uncommon. Consequently, the application of data processing methodologies similar to those employed for surface imagery is not yet justified. At this stage, the underlying degradation mechanisms are therefore hypothesized with such a limited database, and the proposed classification framework should be regarded as a prospective schema intended to support future investigations as the structures mature and distress manifestations become more prevalent. The primary objective of introducing 3D GPR is to improve the understanding of the specific phenomenology associated with the RUP-FS structure.

This combined approach enhances the accuracy and efficiency of RUP-FS diagnostics while reinforcing sustainable maintenance practices. We structured our databases according to the type of acquisition device that is used (Fig. 2), categorizing data as follows:

- **UAV:** Detection and classification of full slabs with a porous concrete wearing course, half/quarter slabs (located along the edges and corners of the structure), and

concrete cobblestones.

- **UGV:** Detection and classification of structural anomalies, including uneven slabs (vertical misalignment between slabs), damaged slabs, pollution colouring, seal spacing/unfilled joints.
- **Stepped-Frequency 3D GPR:** Discrimination of slab conditions, distinguishing between healthy slabs, slab-specific defects, and external issues such as excavation base collapses.

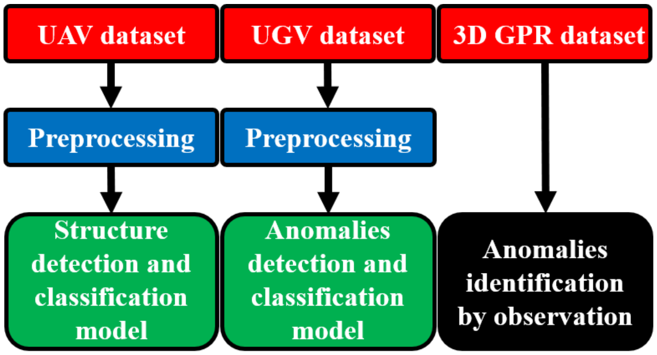

Fig. 2. Workflow illustrating the multi-technology processing for RUP-FS structure detection and anomaly classification.

The objective of this manuscript is to introduce a feasibility study based on limited surface and subsurface datasets acquired on an innovative pavement structure. To carry out this research, the article is structured as follows: Section 2 describes the applied methodology for processing the collected data along with the equipment used, leveraging it for detection, classification, and analyzing surface-level anomalies. Section 3 presents the experimental test sites, including the fatigue carousel at Gustave Eiffel University (Nantes campus) and the open-road site located in downtown Nantes (France) and a detailed overview of key anomalies. Section 4 presents results and discusses, while Section 5 concludes the paper with outlooks.

II. Methods

A. Set-up

To enable the discrimination of the observed structure with UAV, the detection and classification of anomalies with UGV, and the observation of subsurface conditions using 3D GPR, it is essential to collect a comprehensive multi-technical/multi-physical dataset. This large volume of annotated and preprocessed data allows for the training of deep learning algorithms, specifically the YOLOv4 architecture, in an *ad hoc* manner.

Data acquisition is conducted at two experimental sites (Fig. 3) using multiple devices, depending on the specific requirements:

- **Aerial data from the fatigue carousel (site 1:** Images are captured using the *DJI Air 2S* UAV (Fig. 4a) at different heights (5, 10, and 15 m) and from various angles. Manual flights are planned and executed with adjustments to ensure sufficient image overlap. Pictures have a resolution of $5{,}472 \times 3{,}078$ pixels. During data collection, adverse weather conditions, including light rain and strong gusts of wind, prevented certain maneuvers from being performed for safety reasons. In total, 118 images are acquired across all altitudes [32];

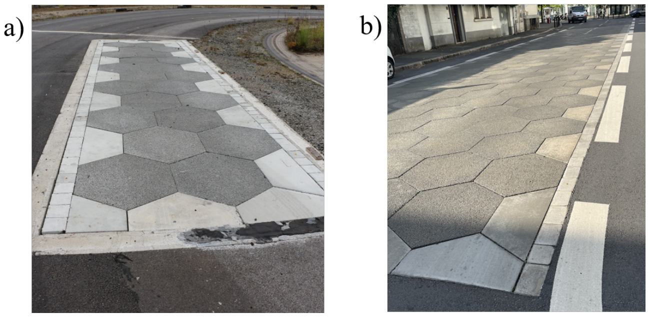

Fig. 3. RUP-FS test sites: a) site 1: Fatigue carousel RUP-FS test structure; b) site 2: Allier Street RUP-FS demonstrator in Nantes city center, France.

- **Aerial data from Allier street demonstrator (site 2):** Images are taken using an HD smartphone camera with selfie stick (Fig. 4b). Pictures have a resolution of $12{,}000 \times 5{,}400$ pixels. Only 5 images are acquired at 3 m height to test the YOLOv4 model;
- **Ground-level data simulating the HERON UGV (all sites):** Images are taken at a maximum height of 1 m above the ground using an HD smartphone camera (Fig. 4b). The angle of capture varied, as did lighting and contrast. Pictures have a resolution of $4{,}000 \times 3{,}000$ pixels. Data are collected under ideal weather conditions, with constant sunlight and dry surfaces. Each slab is photographed with sufficient overlap, and additional images are taken from different angles. A total of 59 images are collected at the fatigue carousel (Site 1) to train and validate the model and 44 images on Allier street (Site 2) to test the model;
- **Geolocated multi-channel microwave electromagnetic data (all sites):** Data is obtained using a network of ground-coupled antennas, specifically the KONTŪR DXG1820 (Fig. 4c), operating over a broad frequency range [0.2 - 2.98 GHz]. At the fatigue carousel, the datasets are collected on a dry surface RUP-FS structure. Although the most recent rainfall, about a week before, has been absorbed by the draining surface concrete, it may still affect the plain concrete on the different elements of the RUP (surrounding concrete cobblestones, half/quarter and full slabs). A second dataset is collected on Allier street under dry weather conditions, with no rainfall events for more than a week prior to acquisition. A longitudinal acquisition step of 1 cm is selected. Two time windows are used: 25 ns, to achieve the highest possible resolution in the near-surface region for analyzing the RUP-FS structure itself, and 35 ns, to enable the identification of potential artifacts beneath structure, such as subgrade condition, buried utility crossings, and other extrinsic factors to the RUP-FS.

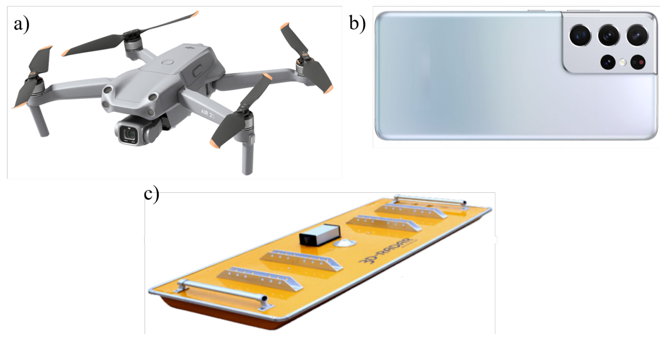

Fig. 4. Devices used for data collection: a) DJI 3S (UAV); [33] b) Smartphone HD camera [34]; c) Ground-coupled antennas Kontūr 3D radar [35]

B. Data augmentation

Studied localized surface defects in RUP-FS exhibit strong visual saliency and limited intra-class variability at the image scale considered in this study. For such visually distinctive anomalies, the performance is related to data variability and acquisition conditions (diffuse illumination due to cloudy brightness, surface moisture–induced halos, local texture heterogeneity), which may contribute to the occurrence of false positives. Consequently, the collected raw surface datasets may be considered incomplete, with a limited number of training images (118 aerial images and 59 ground-level images). Thus, to increase dataset variability, acquisitions were conducted across different seasons. This resulted in observable differences between the two test sites, including the presence of localized wet areas and partial shadowing caused by surrounding trees. As a result, data augmentation is necessary [36, 37]. Augmentation process generated additional data through geometric transformations (such as rotation, reflection, and scaling) and HSV color space variations (Hue, Saturation, Value). These preprocessing steps increased the number of training samples, improving model efficiency while ensuring a balanced dataset size [38, 39, 40, 41].

C. YOLOv4 algorithm

As discussed in Section I, the YOLOv4 (You Only Look Once version 4) architecture is selected. Introduced in 2020 [28], YOLOv4 is a real-time object detection algorithm that balances accuracy and computational efficiency. Designed for processing images and video streams with minimal computational load, YOLOv4 is widely applied, including for detecting defects in concrete [42] and anomalies in transport infrastructures [43]. The modular YOLO architecture consists of three main components (Fig. 5):

- **Backbone:** Extracts complex features from input images while optimizing accuracy and speed.
- **Neck:** Fuses extracted features to enhance object detection robustness across different scales, using multi-scale pooling layers.
- **Head:** Positions objects within images and predicts their classes using *anchor boxes* for localization.

Although YOLOv4-tiny is not the most recent deep learning architecture, it is selected because it fits the specific requirements of RUP-FS surface anomaly detection and classification.

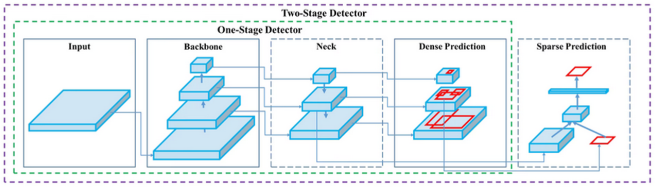

Fig. 5. YOLOv4 architecture [28]

The targeted elements (UAV imagery) and anomalies (UGV imagery) present strong visual contrast and relatively low structural complexity, so a lightweight convolutional model is sufficient to extract the relevant features. Our experiments indicate that detection performance is mainly influenced by data quality and acquisition variability rather than by network depth. In addition, the low computational cost of YOLOv4-tiny enables real-time inference and facilitates integration on mobile inspection platforms [27, 44, 45, 46]. Using more complex architectures would certainly significantly improve global performance; it would also entail substantial computational and integration costs. Objective is not to benchmark state-of-the-art models but to develop a deployable solution for large-scale road surface monitoring. Consequently, the choice of YOLOv4-tiny is motivated by the deployment context targeted in this study, namely real-time inference on embedded and computationally constrained platforms. While recent models such as YOLOv8 can achieve higher detection accuracy, YOLOv4-tiny, in addition to being sufficient for this feasibility study, offers a more favorable trade-off between accuracy, latency, model complexity, and computational cost, which is more consistent with the requirements of edge deployment.

For this research work, YOLOv4-tiny model is trained and validated on a Dell Precision T7910 workstation equipped with an Intel®Xeon®E5-2630 v3 CPU (2.40 GHz, 8-Core), 128 GB DDR4 RAM, and an NVIDIA Quadro K2200 4GB GPU. Training is performed with a *Mini Batch Size* of 16 over 20 epochs (with model convergence observed around the tenth epoch). The image resolution is set to 608×608. The *Adam* optimizer is chosen, leveraging adaptive gradient descent techniques (AdaGrad) and root mean square propagation (RMSProp) [47]. Each model parameter has its own learning rate, which adapts throughout the training process. The model has low memory resource requirements and incorporates the Efficient Layer Aggregation Network (ELAN) concept, enabling efficient convergence even in the presence of slight gradient variations.

However, due to the very high resolution of the collected images, we decided to adopt a more compact version of the YOLOv4 model, namely YOLOv4-tiny. Both YOLOv4 and YOLOv4-tiny share the same philosophy of real-time detection, but the tiny version enables faster inference on embedded systems (ranging from 4.5× faster [48] to up to 10× faster [49]) at the cost of reduced accuracy. Since the primary objective of the HERON project is to achieve rapid detection, we chose the YOLOv4-tiny architecture for the remainder of this study. Even today, the YOLOv4-tiny remains an excellent balance between speed, simplicity, and resource

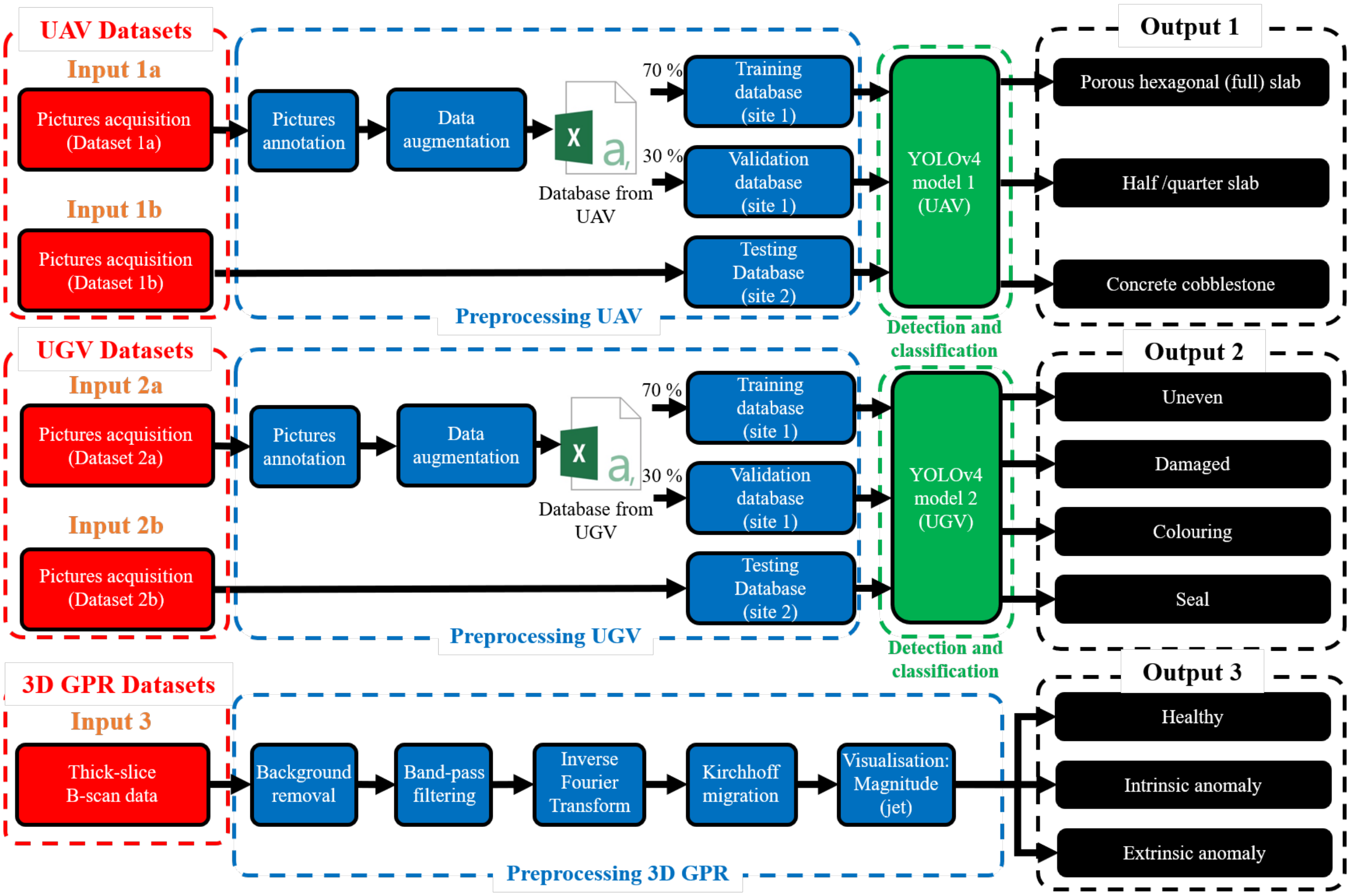

Fig. 6. Full workflow illustrating multi-technique data acquisition, preprocessing methods, and surface/subsurface anomaly detection and classification (with training/validation on site 1 and testing on site 2)

usage. Moreover, another component of the HERON project applies the same methodology to detect and classify surface defects on conventional pavements (e.g., potholes, cracks, missing road markings, and the presence of obstacles or traffic cones) [26]. Here, due to the controlled prefabrication process and the repeatability of RUP-FS production, the core of the algorithmic model lies in the detection and discrimination of the distinctive geometric pattern of hexagonal slabs. Furthermore, the macrotexture of the surface porous concrete can be distinguished from the dense hydraulic concrete used in half/quarter-slabs, as well as from the cobblestone border surrounding the structure. Finally, urban pollution and the varying environmental conditions during image acquisition constitute key factors for verifying the feasibility and assessing algorithmic YOLOv4-tiny performance using appropriate metrics.

D. Metrics

To effectively assess the performance of the object detection model, standard metrics are employed, including *Precision* (eq. 1), *Recall* (eq. 2), *F1-score* (eq. 3), and *mean Average Precision (mAP)* (eqs. 4 and 5). The *mAP50* represents the average precision computed at a fixed *Intersection over Union (IoU)* (e.g. the overlap between a predicted bounding box and a ground-truth bounding box) at a 0.50 threshold.

$$\text{Precision} = \frac{TP}{TP + FP} \tag{1}$$

$$\text{Recall} = \frac{TP}{TP + FN} \tag{2}$$

$$\text{F1-Score} = \frac{2 \times TP}{2 \times TP + FP + FN} \tag{3}$$

$$AP = \int_0^1 p(r)\, dr \tag{4}$$

$$mAP50 = \frac{1}{N} \sum_{k=1}^{N} AP_k \tag{5}$$

where TP represent the True Positive, FP the False Positive, FN the False Negative, $p(r)$ the precision-recall curve and N the number of classes, respectively.

Figure 6 presents the complete methodological workflow. As input, the UAV-acquired imagery is divided into two independent subsets: Input 1a, dedicated to model training/validation, and Input 1b, reserved for testing. The manual preprocessing stage consists of annotating each image to identify 3 classes corresponding to the structural components of the RUP-FS system (concrete cobblestones, half/quarter

and full slabs). Anchor boxes are used to discriminate each element and extract their spatial coordinates. Data augmentation is subsequently applied to the ground-truth bounding boxes prior to their use as input to a first YOLOv4 model, specifically configured for the detection and classification of surface components from UAV-based imagery.

An analogous methodology is implemented for the UGV-acquired dataset. The ground-level images are similarly partitioned into two subsets (Input 2a and Input 2b) for training/validation and testing, respectively. In this case, preprocessing targets 4 distress classes (unevenness, damage, pollution colouring and sealing) identified as the primary surface anomalies. Following annotation and data augmentation, the corresponding ground-truth bounding boxes serve as inputs to a second YOLOv4 model, dedicated to the detection and classification of surface distresses from UGV imagery.

Regarding the Stepped-Frequency 3D GPR dataset (Input 3), as previously noted, the recent construction of the experimental sections resulted in a very limited number of subsurface anomalies, thereby precluding large-scale detection and classification. Nevertheless, qualitative thick-slice visualization enables the anticipation of contrasts between structurally healthy conditions and areas showing elevated gradient permittivity (revealing subsurface defects).

Finally, it should be highlighted that the three datasets (UAV, UGV, and 3D GPR) are intentionally processed independently, as the objective is to show the discrimination capability of each platform under its own acquisition conditions. The coordination framework of the European HERON research project first relies on UAV-based detection to distinguish RUP-FS structures from conventional pavements (initial processing using the YOLO model) [32]. The UAV then transmits a geolocated intervention request to the UGV, which autonomously navigates to the target area to acquire high-resolution images and perform real-time processing (second stage using the YOLO model) [50]. In contrast, the 3D GPR system is not yet automated and currently requires human intervention. Therefore, this work does not fall within a multimodal fusion framework but rather constitutes a comparative analysis across different sensing platforms, ensuring that no cross-influence occurs during model development or use, and thereby adhering to the principle of real-time coordination. The main goal of this manuscript is to address the issues of step number *2. Defect detection* of Figure 7)

The work presented here focuses on the methods developed in Work Package 3 (WP3) of the HERON project (*AI-based Algorithms and Tools for Recognition, Classification and Localization of Points of Interest*) [51]. The full system integration and its operational deployment are addressed at the overall project level and fall outside the scope of this manuscript. Consequently, this study introduced the methodological validity of the subsystem rather than providing a final demonstration of the complete system.

After detailing the data acquisition systems and the algorithmic methodology used for their processing, Section III presents the experimental sites and the main surface and subsurface defects identified.

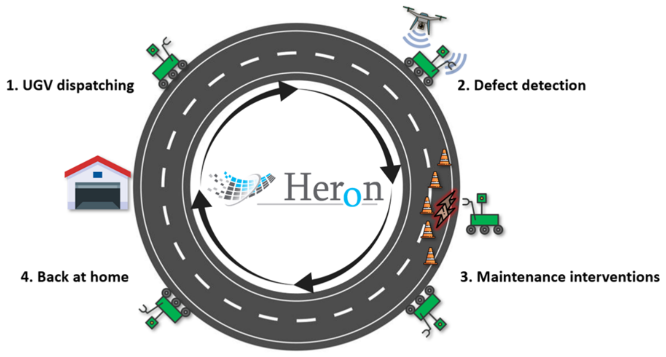

Fig. 7. HERON: Integrated concept for road diagnosis and treatment [20]

III. EXPERIMENTAL SETUP

A. Site 1: fatigue carousel at Gustave Eiffel University (Nantes/France)

The fatigue carousel at Gustave Eiffel University (Nantes, France) is in operation for over 40 years and serves as a unique facility for accelerating the aging of full-scale pavement structures while studying their long-term behavior under repeated loading [13]. The system consists of four rotating arms equipped with tandem or tridem wheels that simulate heavy vehicle loads, operating at speeds of up to 100 km on a circular track with a diameter of 120 m (Fig. 8).

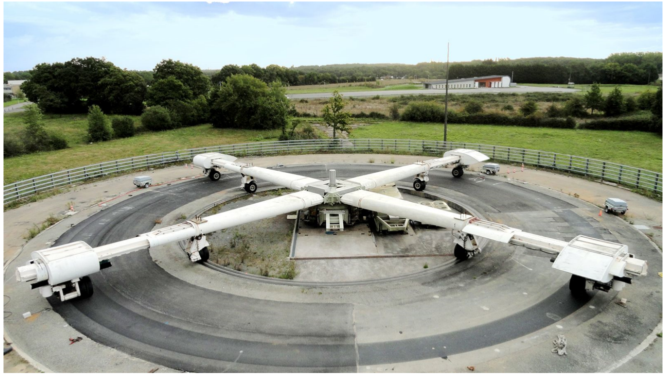

Fig. 8. Fatigue carousel at Gustave Eiffel University, Nantes (France) [13]

As part of the I-Street project, a Removable Urban Pavement with Functionalized Surface (RUP-FS) is installed on the fatigue carousel to conduct mechanical and functional performance tests (Fig. 3a). The test structure measures 8.35 m in length and 2.31 m in width, comprising 39 slabs of 23 cm thickness and 46 cm edge length. The setup includes 15 half-slabs and 2 quarter-slabs made of plain concrete, while the 22 full slabs consist of a 19 cm plain concrete base topped with a 4 cm porous concrete surface layer (used for water drainage). Additionally, cubic concrete cobblestones are longitudinally integrated into the structure permitting dismantling. Joint filling with sand is targeted between each slab. The objective is to evaluate the mechanical performance, acoustic properties, and drainage capacity of the slabs.

To simulate heavy vehicle traffic, accelerated fatigue tests are conducted, replicating 200,000 rotation cycles over two

months. The test axle, equipped with dual wheels, is loaded to 65 kN, simulating a daily traffic volume equivalent to 45 to 91 buses per direction over a 20 years' service life. The results confirm the mechanical resistance and skid resistance of the structure, demonstrating its adaptability to real-world traffic conditions [9].

B. Site 2: Allier Street in Nantes City Center

In addition to the fatigue carousel tests, a full-scale demonstrator is built on Allier Street in downtown Nantes (France) and commissioned in early 2022. This segment, measuring 31.28 m in length and 3.68 m in width, covers two traffic lanes adjacent to a dedicated bus lane (Fig. 3b). The structure consists of 345 slabs, each 23 cm thick and 46 cm edge width. Among them, 57 half-slabs and 2 quarter-slabs are made of plain concrete, while 286 full slabs feature a 19 cm plain concrete base topped with a 4 cm porous concrete layer (used for water drainage). Rectangular concrete cobblestones are also incorporated to ensure longitudinal confinement and to permit deconstruction. This demonstrator, located in a high-traffic urban environment, undergoes daily exposure to intensive loads from passenger vehicles, heavy trucks, and public transport. This second site also benefits from exposure to urban pollution and the presence of spurious objects (e.g., cigarette butts, crushed cans), partial shadowing caused by surrounding trees, etc. These factors differ significantly from the controlled conditions of the fatigue carousel, introducing elements that may interfere with automated data processing. In addition, the joint filling with sand procedure differs from that used on the fatigue carousel, as it is applied over the entire surface and subsequently swept to fill the joints.

C. Challenges

For effective defect detection and geolocation, it is crucial first to identify RUP-FS structures within conventional infrastructure. Identification relies on recognizing specific geometric features, such as full slabs (porous hexagonal concrete surface layer), half/quarter slabs, and perimeter concrete cobblestones (Fig. 9a). This step also facilitates slab and paver counting, helping in construction monitoring and infrastructure inspection.

- **Porous hexagonal (full) slab:** Primary structural component, incorporating a permeable wearing course to ensure surface drainage.
- **Half/quarter slab:** Concrete element located along the perimeter of the structure, ensuring edge confinement.
- **Concrete cobblestone:** Peripheral paving stone units forming a continuous boundary that frames and laterally confines the overall structure.

These 3 classes are identified as 'Output 1' in the flowchart presented in Figure 6.

Then, implementation process, traffic-induced mechanical stresses, and environmental variations contribute to the emergence of various structural and functional defects. Key surface defects that significantly impact infrastructure safety and usability include:

- **Unevenness:** Vertical misalignment between slabs, often indicative of differential settlement, excessive joint clearance, or failure of the concrete interlocking keys. This specific type of defect detection can complement longitudinal and transverse profile measurements (not performed in this study) and support structural maintenance, particularly by addressing the risk of collapse. (Fig. 9b),
- **Damage:** Edge spalling can increase rolling noise. These defects are often caused by impacts during installation. Additionally, localized surface detachments may occur due to porous concrete deterioration from impacts, freeze-thaw cycles, or mechanical wear (Fig. 9c),
- **Colouring:** Surface pollution (e.g., oil stains, tire marks, chewing-gum) primarily affect aesthetic appearance but may occasionally alter skid resistance (Fig. 9d),
- **Sealing:** Reduction in interlocking between slabs, leading to joint widening and the progressive displacement of filling sand into the underlying gravel bed (Fig. 9e).

These 4 classes of anomalies are identified as 'Output 2' in the flowchart presented in Figure 6. The annotation scheme was defined based on a study conducted after controlled aging of the structure on the fatigue carousel, equivalent to 20 years. The surface anomalies observed at that stage were selected as representative of real-world deterioration. Subsequently, after 3 years of in-service aging on the Allier Street demonstrator, the same types of degradation were identified and ultimately retained for the final detection and classification model.

As part of the HERON project, this initial detection and classification are performed using UAV. This preliminary phase is essential for providing precise guidance to the HERON UGV, which is responsible for detailed defect detection and analysis using advanced image recognition and classification algorithms.

Beyond surface defect analysis, multi-channel microwave electromagnetic devices provide in-depth subsurface estimations, detecting anomalies not visible through UAV, UGV or visual inspections. These anomalies may stem from previously identified surface defects, such as spalling or joint widening, which could accelerate deeper structural pathologies. Conversely, certain subsurface defects may contribute to surface-level issues, such as misalignment. The identified classes fall into three main categories:

- **Healthy slab:** No anomaly detected (This class could potentially be subdivided into two subclasses: with or without metallic fibers),
- **Intrinsic to the RUP-FS structure:** Breakage of concrete interlocking keys, drainage malfunctions due to clogged vertical drains beneath the porous concrete overlay, etc.
- **Extrinsic to the RUP-FS structure:** Settlement of the excavatable sub-base, water accumulation within the gravel bed beneath the slabs, increasing structural instability risks, etc.

These 3 classes are identified as 'Output 3' in the flowchart presented in Figure 6.

These subsurface analyses are critical for establishing pre-

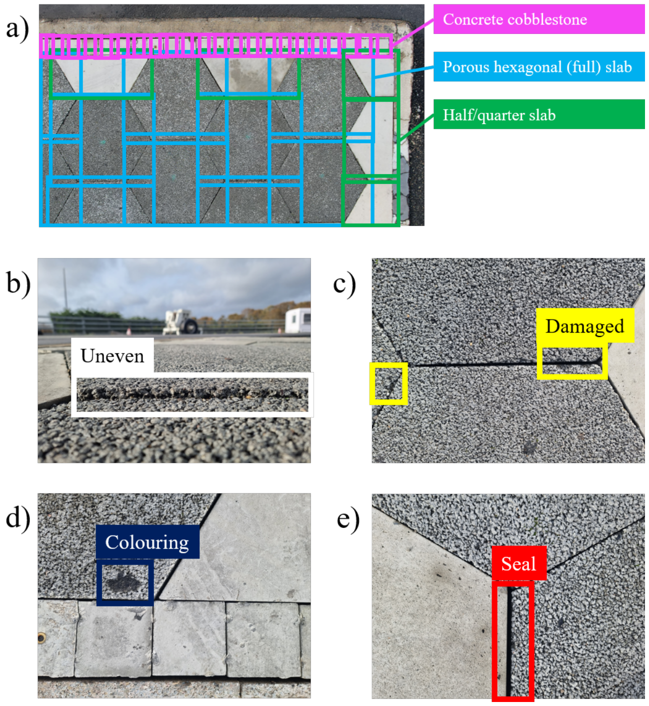

Fig. 9. Identification of RUP-FS structure and defects: a) Uneven; b) Damaged; c) Colouring; d) Seal; e) Identification of different geometries and surface materials.

cise correlations between surface defects and deeper structural pathologies. They also guide preventive and corrective maintenance interventions, ultimately optimizing the durability of RUP-FS structures.

In Section IV, datasets are segmented based on hardware and experimental zones. Data is annotated and preprocessed before applying YOLOv4-tiny (following methodology presented in Fig. 6). The first case examines UAV-based structural element detection and classification, the second focuses on surface anomalies using UGV images, and the final study compares subsurface conditions to differentiate healthy slabs from those with intrinsic or extrinsic defects. Regarding the subsurface investigation, only a qualitative and non-automated interpretation was conducted. Unlike surface imagery acquired from UAV and UGV devices, the 3D GPR signals showed a very low proportion of anomalies, regardless of depth. Consequently, only a projection of relevant classes that could be considered for future research has been proposed. At this stage, the development of an automated approach would be premature and potentially biased due to the insufficient representation of anomalies.

IV. Results and Discussion

A. *UAV results analysis*

118 UAV images from site 1 are used as input. As discussed in Section II-B, this number of images is insufficient to achieve optimal performance. Data augmentation increased the dataset to 3776 UAV images, incorporating geometric transformations (such as rotation, reflection, and scaling) as well as variations in the HSV color space (Hue, Saturation, Value). Among these, 2643 images (approximately 70 %) are used for training. The different elements are delineated using anchor-based annotations. The objects of interest are labeled using ground-truth bounding boxes (full slabs, half/quarter slabs, and concrete cobblestones) (Fig. 10). Table I reports the number of instances for each object class before and after data augmentation. These ground-truth bounding boxes constitute the input targets of the detection model during training. By design, a RUP-FS structure inherently contains more cobblestones than slabs. The class distribution in our dataset reflects the natural prevalence observed in the target application. In this context, class imbalance is not an artifact but an intrinsic property of the task. To address this, we assessed the model using per-class metrics (as presented in II-D) and recall-oriented measures rather than relying only on overall accuracy, which is known to be misleading in imbalanced settings. In this study, the use is intentionally aligned with real-world conditions rather than applying rebalancing techniques, which would distort the target distribution by artificially overrepresenting certain classes and, in turn, reduce the effective diversity of the dataset.

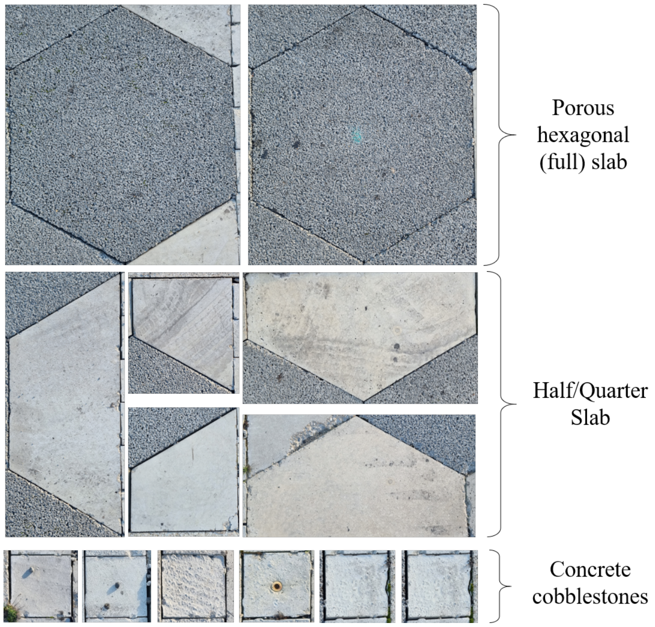

Fig. 10. Example of UAV Input ground-truth bounding boxes

TABLE I
NUMBER OF UAV INPUT GROUND-TRUTH BOUNDING BOXES

Class	Number of bounding boxes before data augmentation	Number of bounding boxes after data augmentation
Full slab	2,119	67,808
Half/Quarter slab	1,521	48,672
Concrete cobblestone	9,796	313,472

Figure 11a illustrates an example of the feasibility of the detection and classification results of the RUP-FS structure, thanks to orthogonal UAV-based images, using the YOLOv4-tiny neural network architecture on the fatigue carousel. In this case, the model is trained on data from the fatigue

a) b)

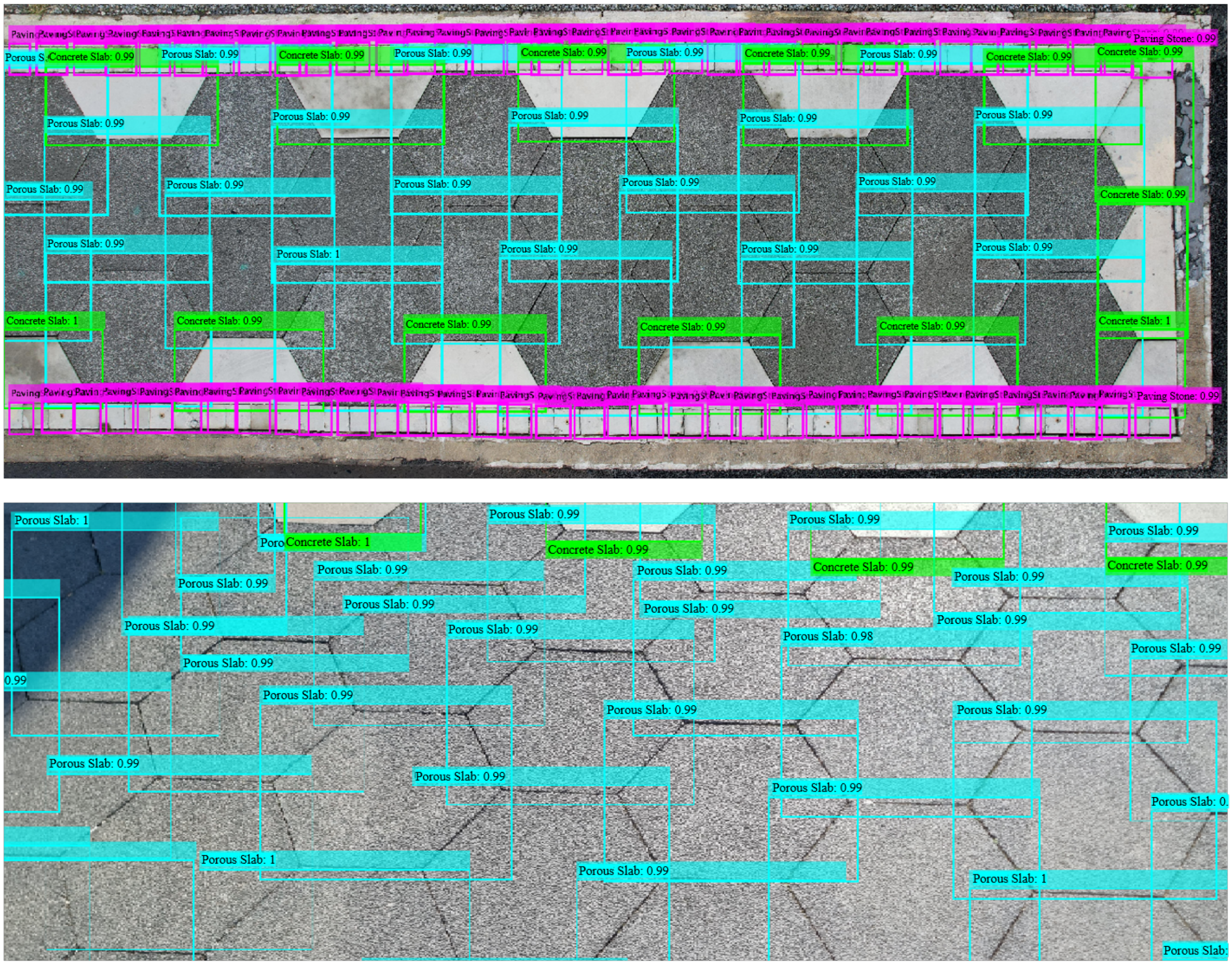

Fig. 11. Representative YOLOv4-tiny outputs from UAV imagery (cyan: full slab, green: plain slab, magenta: concrete cobblestone) : a) On the fatigue carousel - Gustave Eiffel University (Nantes Campus); b) On Allier street

carousel (Site 1) after training. Thus, based on the number of boxes in each class it is possible to classify and count the three different elements of the RUP-FS structure: full slab, plain half/quarter slab, and concrete cobblestone. In Fig. 11b, acquired from Site 2, the YOLOv4-tiny model further illustrates the feasibility of detecting and classifying the different structural elements, despite the different acquisition altitude (approximately 3 m) and the image distortion (anamorphosis) induced by the deliberate tilt of the acquisition system.

The results Fig. 11b are derived from the test dataset, which consists solely of photos taken at Site 2 (Allier street), with training/validation conducted exclusively on Site 1. It should be noted that the images collected at Site 2 are not included in the training process, neither for the UAV-based data nor for the UGV-based data. Consequently, datasets are split into training/validation sets (collected at Site 1) and a test set (applied to Site 2). Despite the limited size of the dataset, this separation suggests promising potential for future generalization to other environments.

Regardless of the site examined, the YOLOv4-tiny algorithm is able to identify and discriminate between the various surface elements making up the structure. In the case of inbalanced data (Table II), the model demonstrates an accuracy (prediction rate) and recall (positive instances) greater than 99 % across all classes. These results are confirmed through the F1-Score and mAP50 (mean Average Precision at 50 %) indicators. However, care should be taken regarding the potential introduction of bias due to the non-exhaustiveness of the dataset. Then, it is important to underline that the UAV acquisition campaign was conducted only once on a dry RUP-FS structure isolated from urban pollution, which may have reduced learning-related errors due to the absence of disturbing factors such as localized moisture areas, unwanted objects, shadow projections, and other environmental artifacts. The results obtained in the case of rebalanced data (Table III) show no significant changes. This can be explained by the strong geometric separability between classes. At this level, the YOLOv4-tiny algorithm is able to converge toward a near-optimal solution regardless of whether the data are imbalanced or balanced. Therefore, dataset rebalancing is not strictly necessary for the detection and classification of structural elements in a RUP-FS structure.

It can be observed that the shape of the concrete cobblestones differs between Site 1 and Site 2, being cubic and

rectangular, respectively. Despite this variation in shape, the impact on classification performance for this category remains minimal. This can be attributed to the strong geometric contrast with other shapes encountered during training (full slab, half/quarter slab and concrete cobblestone) and data augmentation including HSV color space variation. Different performance metrics are consistent with one another and suggest the potential of the YOLOv4-tiny architecture for accurately classifying surface elements within the scope of this feasibility study.

TABLE II
PERFORMANCE METRICS OF THE YOLOV4-TINY MODEL FOR UAV APPLICATIONS (SITE 1 IMBALANCED DATA VALIDATION RESULTS)

Umbalanced lass	Precision	Recall	F1-score	mAP50
Full slab	99.9 %	100 %	100 %	99.5 %
Half/Quarter slab	99.7 %	98.2 %	98.9 %	99.5 %
Concrete cobblestone	99.8 %	99.1 %	99.4 %	99.5 %
All UAV classes	99.8 %	99.1 %	99.4 %	99.5 %

TABLE III
PERFORMANCE METRICS OF THE YOLOV4-TINY MODEL FOR UAV APPLICATIONS (SITE 1 REBALANCED DATA VALIDATION RESULTS)

Rebalanced class	Precision	Recall	F1-score	mAP50
Full slab	99.8 %	99.8 %	99.8 %	99.3 %
Half/Quarter slab	99.5 %	98.9 %	99.2 %	99.3 %
Concrete cobblestone	99.7 %	98.9 %	99.3 %	99.3 %
All UAV classes	99.6 %	99.2 %	99.4 %	99.3 %

The first part of this research confirms the feasibility of identifying the various structural elements at different heights (5, 10, and 15 m) from the UAV for classification. Following the guidelines established by the HERON project, we can count the number of bounding boxes per class and, consequently, the number of elements to facilitate construction site monitoring. Additionally, it is possible to geolocate a RUP-FS structure to guide the UGV toward the area for inspection and surface anomaly detection. The following paragraph focuses on the data collected on the ground by the UGV.

As observed during the data acquisition phase, the UAV's spatial resolution allows clear discrimination of the different elements composing the structure. However, the detection and classification of surface defects remain insufficient for a precise analysis. For this reason, an UGV is used in the subsequent stage of this study.

B. UGV results analysis

59 UGV images are used as input. As noted in Section II-B, this number is likewise insufficient to ensure optimal performance. Data augmentation expanded the dataset to 1888 UGV images, including geometric transformations (such as rotation, reflection, and scaling) and HSV color space variations (Hue, Saturation, Value). Of these, 1322 images (approximately 70 %) are used for training on Site 1. The different elements are delineated using anchor-based annotations. The objects of interest are labeled using ground-truth bounding boxes (Uneven, Damaged, Colouring, Seal) (Fig. 12). Table IV reports the number of instances for each object class before and after data augmentation. These ground-truth bounding boxes constitute the input targets of the detection model during training.

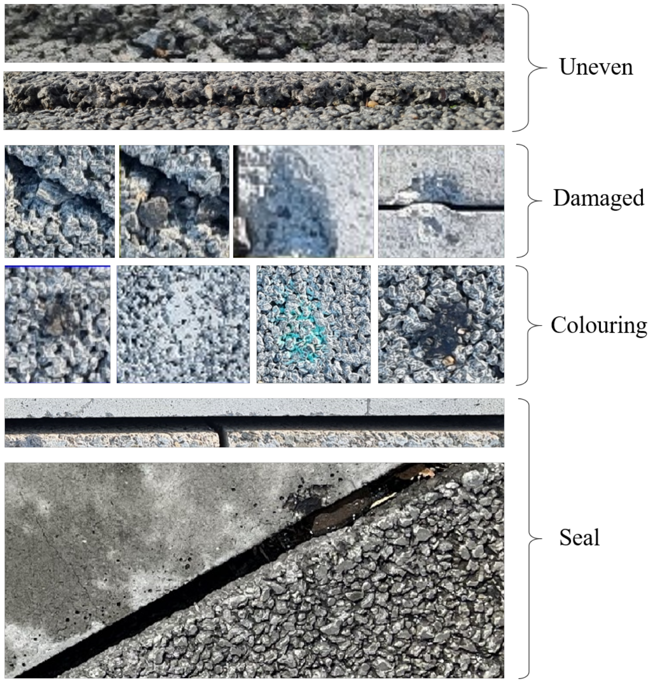

Fig. 12. Example of UGV Input ground-truth bounding boxes

As noted previously, a class imbalance can be observed, with the *Damaged* class being overrepresented relative to the others. The dataset imbalance was intentionally preserved because it reflects the operational distribution of the target problem; consequently, we report per-class and recall-sensitive metrics to ensure that minority-class performance is properly assessed. Indeed, the defects constituting both the training dataset (controlled aging on the fatigue carousel) and the test dataset (in-service aging on Allier Street) are exhaustive and representative of real degradation under realistic conditions and inter-class proportions. This distribution of anomaly occurrence was therefore maintained to ensure that the performance of minority classes is accurately represented without artificially overrepresenting them.

TABLE IV
NUMBER OF UGV INPUT GROUND-TRUTH BOUNDING BOXES

Class	Number of bounding boxes before data augmentation	Number of bounding boxes after data augmentation
Uneven	70	2,240
Damaged	550	17,600
Colouring	99	3,168
Seal	56	1,792

The results presented in Figure 13 illustrate the surface anomaly classification outcomes on the RUP-FS at Site 1 (fatigue carousel) through four representative examples: Damaged in Figure 13a, Colouring in Figure 13b, Uneven in Figure 13c, and Seal in Figure 13d. The model validation performance metrics are displayed directly adjacent to the detected anomalies and show the results achieved.

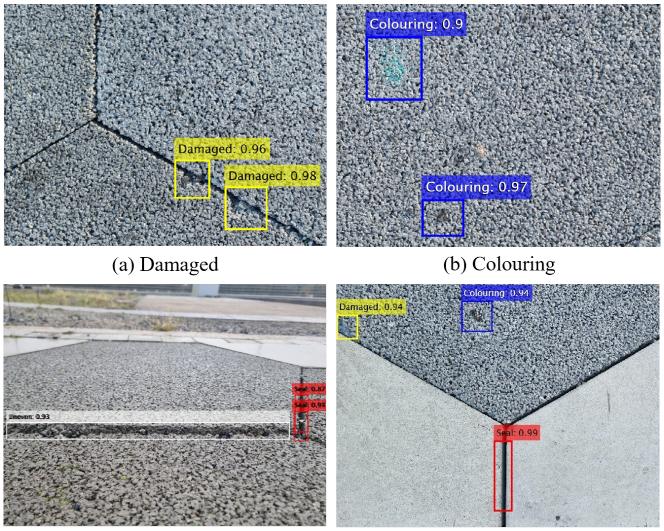

(a) Damaged (b) Colouring

(c) Uneven and seal (d) Damaged, colouring and seal

Fig. 13. Representative YOLOv4-tiny Outputs from UGV Imagery of the fatigue carousel (white: uneven between slabs, yellow: damaged, blue: colouring, red: too large seal and/or unfilled joint)

The results (Fig. 14) are derived from the test dataset, which consists solely of photos taken at Site 2 (Allier street), with training/validation conducted exclusively on Site 1. The marked differences between Site 1 and Site 2 in terms of illumination conditions, contrast levels, cast shadows, and the presence of surface moisture (as illustrated in Figure 14b) did not adversely affect anomaly detection and classification performance. This results can be attributed to the precautionary measures implemented during the data augmentation phase, which are designed to account for variability in acquisition conditions.

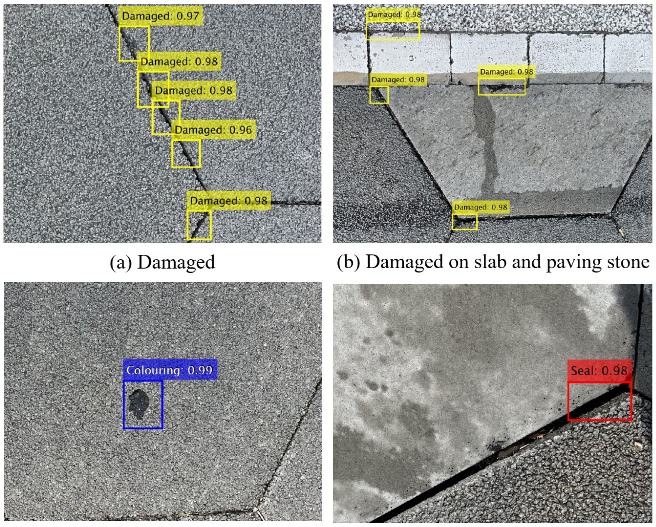

(a) Damaged (b) Damaged on slab and paving stone

(c) Colouring (d) Seal

Fig. 14. Representative YOLOv4-tiny outputs from UGV imagery from the Allier street (yellow: damaged, blue: colouring, red: too large seal and/or unfilled joint)

As with UAV data, YOLOv4-tiny is capable of detecting and classifying defects regardless of the site being studied. Tables V and VI shows the performance across all observed classes.

TABLE V
PERFORMANCE METRICS OF THE YOLOV4-TINY MODEL FOR UGV APPLICATIONS (SITE 1 IMBALANCED VALIDATION RESULTS)

Umbalanced class	Precision	Recall	F1-score	mAP50
Uneven	97.9 %	83.7 %	90.2 %	98.7 %
Damaged	97.5 %	99.9 %	98.7 %	97.4 %
Colouring	95.5 %	99 %	97.2 %	92.1 %
Seal	97.6 %	99.2 %	98.4 %	96.4 %
All UGV classes	95.4 %	96.1 %	96.1 %	96.4 %

TABLE VI
PERFORMANCE METRICS OF THE YOLOV4-TINY MODEL FOR UGV APPLICATIONS (SITE 1 REBALANCED VALIDATION RESULTS)

Rebalanced class	Precision	Recall	F1-score	mAP50
Uneven	97.9 %	90.1 %	93.8 %	98.9 %
Damaged	97.3 %	98.9 %	98.1 %	97.4 %
Colouring	95.5 %	98.9 %	97 %	91.8 %
Seal	97.5 %	99 %	98.2 %	96.3 %
All UGV classes	95.4 %	96.7 %	96.1 %	96.2 %

Similarly, to the classification of structural elements by the UAV (Section IV-A), the model dedicated to classifying anomalies detected by the UGV demonstrates excellent performance. Unlike structural element identification (Section IV-A), where repeating patterns facilitate recognition, ground anomaly classification is inherently more challenging. In this context, the geometry of the damage and the shape and color patterns of contamination stains show significant visual variability, requiring the development of a comprehensive annotated dataset. However, because the surveyed structures are relatively recent and display only a limited number of anomalies, the amount of available data remains restricted. Therefore, data augmentation is required to increase dataset diversity and improve the possibility of future generalization of the detection and classification models. Table V shows, in the case umbalanced data, through the overall mean values of precision, recall, F1-score, and mAP50, that the proposed model achieved accurate recognition across all classes with a very low error rate.

For the *Uneven* class, the recall is lower than for other categories, with $Recall_{Uneven} = 83.7\,\%$, which affects the F1-Score. This can be attributed to the limited availability of representative training data, making it difficult for the model to learn effectively from a small number of samples. Similarly, the precision for the *Colouring* class, although high, is slightly lower than for other classes at $Precision._{Colouring} = 95.5\,\%$. This is also due to the low number of instances in this category. Furthermore, stains may be misclassified as surface detachment and its shadow, which leads them to be classified under the *damaged* class. Comparing now with the case of balanced data, the model performs very well without rebalancing; however, a noticeable improvement in Recall (83.7 % *vs.* 90.1 %) and consequently in the F1-score (90.2 % *vs.* 93.8 %) was observed for the *Uneven* class, with only a marginal impact on the other classes. In the case of defect detection, applying data rebalancing may be beneficial. Indeed,

to mitigate potential driving discomfort for road users, it is particularly relevant to improve the accurate identification of the *Uneven* class. It is also important here to be cautious about the potential introduction of bias due to the non-exhaustive nature of the dataset.

For professional pavement defect detection and risk estimation, it is generally better to keep an unbalanced dataset, as it preserves the real-world distribution of defects. However, this imbalance should be handled during training using techniques such as weighted sampling or targeted augmentation. A fully balanced dataset can improve training stability, but it distorts real occurrence probabilities and reduces the reliability of risk interpretation in operational use.

By integrating data from both the UAV and the UGV systems, a comprehensive assessment of the surface condition of this innovative structure can be achieved. To further enhance these results, subsurface inspection using electromagnetic wave propagation methods presents a promising alternative to gain deeper insights into the underlying structural integrity, which remains invisible to a simple vision without dismantling the structure.

C. Stepped-Frequency 3D GPR results analysis

As discussed previously, surface anomalies can now be detected and automatically characterized. Some of these anomalies may be the result of subsurface defects, such as cracking of the concrete key shown in Figure 1 in the case of *Uneven* class, or ground deformations that affect the spacing between slabs in the case of *Seal* class. The use of microwave wave propagation methods with antennas network allows for the detection of potential dielectric contrasts caused by material changes, thereby ensuring integrity of this innovative pavement.

One of the main challenges in the subsurface inspection of RUP-FS lies in the complex geometry of the slabs, which generates multiple reflections due to edge effects. In addition, the metallic fiber reinforcement embedded in the concrete, designed to reduce the risk of fragile rupture, also introduces multiple reflections (as the metal acts as a perfect reflector). Finally, due to their design, the draining surface layer used as the wearing course in hexagonal full slabs further complicates signal interpretation, as significant dispersion occurs in the presence of water after a rainfall event.

Using the high-resolution Stepped-Frequency 3D GPR with Ultra-Wide Band (UWB) system described in Section II-A, raw frequency-domain data are collected to assess the condition of the subsurface layers. Data are processed in thick-slice, through background removal and band-pass filtering to reduce noise, an inverse Fourier transform for time-domain visualization, and Kirchhoff migration [52]. Knowing the exact geometry of the slab, we can estimate a dielectric permittivity of $\varepsilon_{RUP-FS} = 6$ corresponding to a dry concrete [53]. The combined application of these processing methods enables the use of the degradation state of the RUP-FS structures, whether intrinsic (e.g., cracking within the slab itself, etc.) or extrinsic (e.g., settlement of the soil beneath the RUP-FS, etc.). Figure 15a shows a normalized magnitude planar view (C-scan) of the EM waves propagating through the RUP-FS structure of the fatigue carousel at a depth of 19.3 cm, overlaid on a picture taken by the UAV. In this C-scan cross-section, we observe a noisy signal. The multiple yellow areas are the result of the edge effects caused by the complex geometry of the slabs (EM wave reflection off the edges of the RUP-FS), combined with multiple reflections due to the presence of metallic fibers embedded in their design. Then, the red box zone (in Fig. 15c) reveals a significant gradient permittivity between 14 and 23 cm with a hot point around 19 cm compared to the white box zone (in Fig. 15b), which is taken as a reference.

By examining Fig. 15b, which shows the magnitude of the B-scan after Kirchhoff migration, we observe, within the white box, as in Fig. 15a, scattered low dielectric contrasts (dark blue to green). However, when comparing Fig. 15b (the reference scan of a healthy plain concrete slab) with Fig. 15c, a significant dielectric contrast appears at a depth of roughly 20 cm (in red). As discussed in Section II-A, the heavy rainfall event a week before data acquisition suggests the likely presence of water in the lower part of the slab. This presence of water within the material may therefore bias the estimation of the dielectric permittivity, typically assumed to lie within the range $5 < \varepsilon_{DryConcrete} < 8$, whereas the dielectric permittivity of moist concrete generally varies between $8 < \varepsilon_{MoistConcrete} < 16$ [53]. Further investigations based on the acquisition of permittivity gradients would therefore be relevant. The fact that this slab reveals a localized anomaly before reaching the bottom, unlike the others, supports the hypothesis of an intrinsic defect specific to this one.

At the experimental site 2 in downtown Nantes (Allier street), municipal staff reported a localized subsidence of the structure (Fig. 16). This defect is visible at the surface and may pose a safety risk to users. To investigate the underlying causes of this collapse, a Stepped-Frequency 3D GPR system is used. The objective is to determine whether the defect is intrinsic to the structure or the result of an external event, thereby enabling the implementation of an appropriate maintenance strategy. Following the data acquisition on Allier street, the C-scan (Fig. 17a) shows a pronounced subsurface anomaly at a depth of approximately 20 cm. Recall that this acquisition is performed under dry weather conditions (see Section II-A), which rules out the hypothesis of vertical water transfer as a contributing factor. In this same figure, the region outlined in white can be considered as the reference area (showing no anomaly at this depth).

In contrast, the area outlined in orange (Figs 17a and 17b) corresponds to a group of five full slabs that are cast without metallic fibers inside. Figure 17b compares the full slab groups in the white box (with metallic fibers) and the orange box (without fibers), highlighting the presence or absence of multiple dielectric contrasts previously observed in the fatigue carousel signals. The lack of metallic fibers provides a clearer identification of the internal features of the slab, such as a horizontal contrast (in dark blue) that corresponds to the bottom of the slab (around 24 cm). Additionally, two contrasts corresponding to vertical joints between slabs can be identified at the longitudinal distance 29 m (in dark blue) and 30 m (in green). Theses contrasts cannot be detected in the white box area due to the presence of metallic fibers.

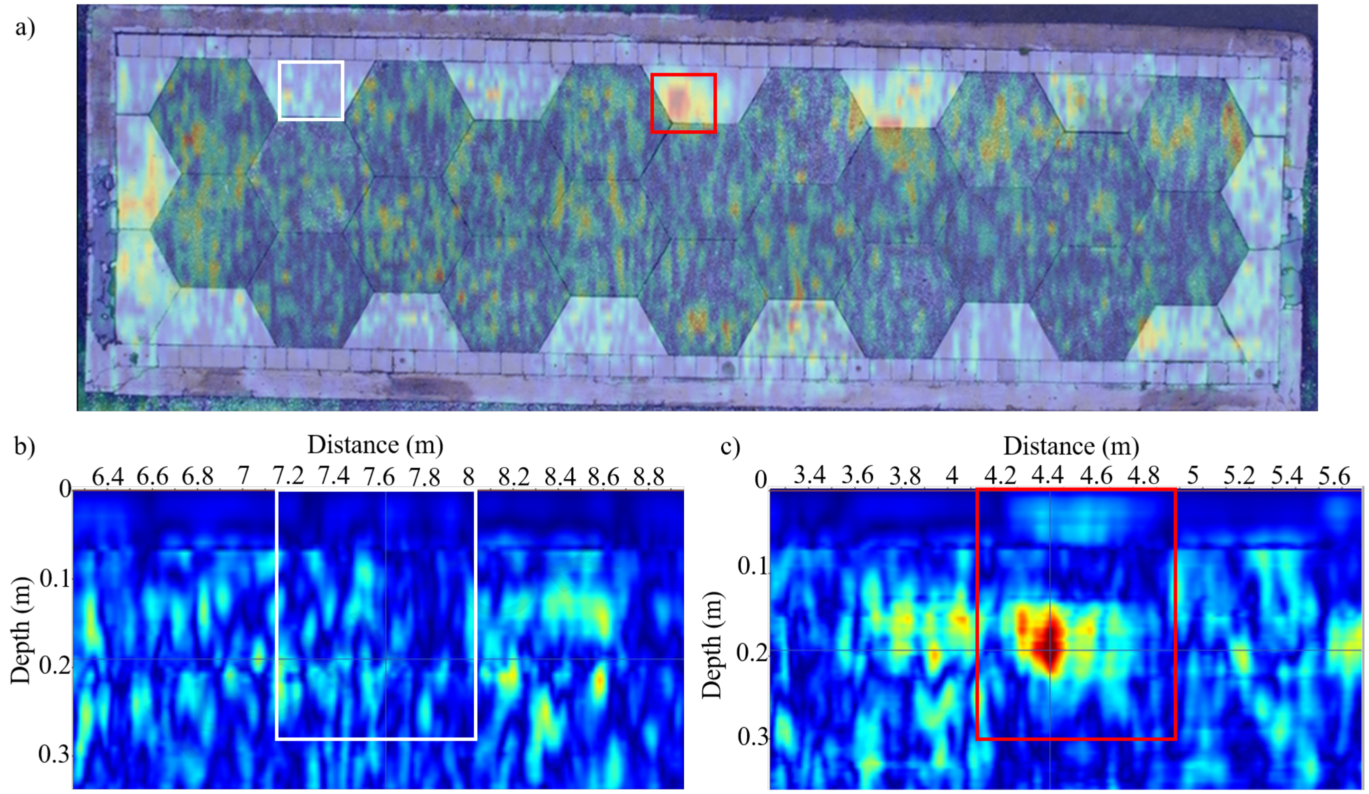

Fig. 15. Stepped-Frequency 3D GPR signal of the fatigue carousel: a) Magnitude C-scan at 19.3 cm depth with Kirchhoff migration superposed on UAV picture ; b) B-scan of a healthy RUP (plain slab); c) B-scan of a RUP (plain slab) with anomaly

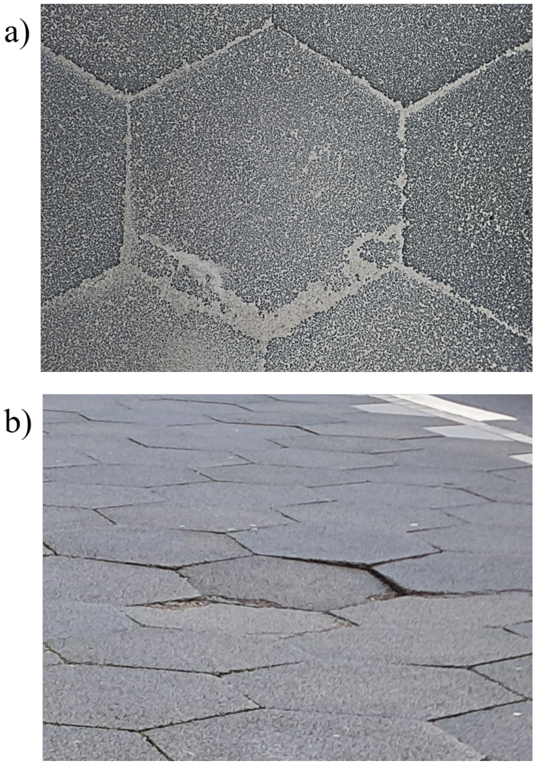

Fig. 16. Localized structural failure in Allier street: a) Top view (Picture taken in June 2024); b) Side view (Picture taken in January 2025)

Finally, Fig. 17c shows significant subsurface dielectric contrasts compared with those observed in the white box in Fig. 17b. A maintenance operation carried out by the Nantes municipal services subsequently confirmed the collapse of a network pipe beneath the structure, which was responsible for the water ingress. This ingress weakened the excavatable sub-base course and, consequently, the RUP-FS structure itself. The use of Stepped-Frequency 3D GPR technology on a RUP-FS structure ultimately makes it possible to observe strong permittivity contrasts despite the complex RUP-FS geometry, to identify potential early-stage damage, and to anticipate major degradations external to the structure, such as the one illustrated here. This defect can therefore be classified as extrinsic.

Through the subsurface phenomenological investigation, the physical interactions between electromagnetic waves and the materials composing this innovative structure were examined. This analysis made it possible to identify potentially relevant features that could pave the way for future automation of detection and classification tasks.

V. Conclusion and Outlook

In conclusion, the innovative Removable Urban Pavement with Functionalized Surface (RUP-FS) structure, conceived and developed by Gustave Eiffel University, has undergone extensive validation tests, including water permeability, mechanical behavior, noises skid resistance, etc. [5].

Within the framework of the European HERON project, the objective is to enable the automatic detection of structural de-

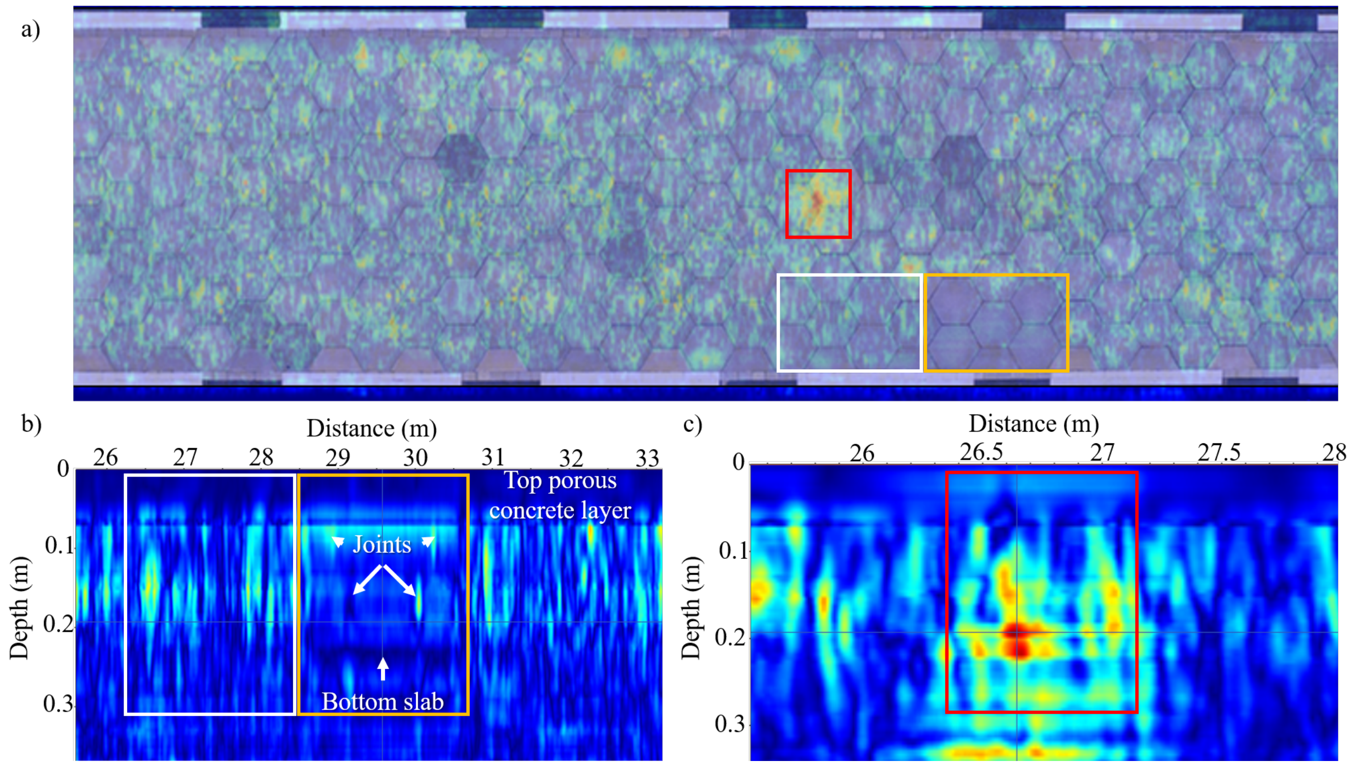

Fig. 17. Stepped-Frequency 3D GPR signal of the Allier street: a) Magnitude C-scan at 19.3 cm depth with Kirchhoff migration superposed on UAV picture ; b) B-scan of a standard healthy RUP-FS (in white) and a healthy non-metallic fiber RUP-FS (in orange); c) B-scan of a standard RUP-FS with anomaly

fects. The approach adopted in this research combines multiple techniques for the characterization of RUP-FS structures. First, a deep learning algorithm based on the YOLOv4-tiny architecture is used to process surface image data collected from different platforms depending on the application requirements (UAV or UGV). UAV-based data acquisition demonstrated the feasibility of the detection and classification of RUP-FS structures at controlled variable altitudes. High-resolution images enabled the discrimination of structural elements (porous and plain concrete hexagonal slabs, and concrete cobblestones) and their differentiation from conventional pavements, achieving a significant mean Average Precision (mAP50) of 99.4 % across all classes (while taking care to account for the potential introduction of bias due to the non-exhaustiveness of the dataset). UGV-based acquisition (using a smartphone to simulate the onboard camera of the robot developed in the HERON project), conducted at one meter above ground level, opened promising perspectives for the detection and classification of surface defects considered critical by road professionals. Four major defect classes (Uneven, Damaged, Colouring, and Seal) were included in the training process, thereby revealing the potential of the proposed approach using the compressed YOLOv4-tiny model which achieved an mAP50 of 96.4 %. These results highlight the performance of YOLOv4-tiny despite the limited size and incompleteness of the training dataset in terms of the cases studied which may introduce potential bias. While more advanced versions of this algorithmic architecture offer only marginal gains in accuracy, they may nevertheless provide improvements in computational efficiency. Although the F1-score and associated performance metrics are highly encouraging, it should be underlined that the current dataset remains insufficiently large to be considered fully representative. Nevertheless, this feasibility study illustrated the viability of the proposed methodology.

The Stepped-Frequency 3D GPR system, exploiting electromagnetic wave propagation, enabled the assessment of the subsurface condition of RUP-FS structures, allowing the distinction between healthy slabs and those exhibiting intrinsic or extrinsic anomalies. The materials used in slab fabrication (with or without metallic fibers) could also be identified through the observation of scattered dielectric contrasts, opening perspectives for future monitoring and optimized maintenance strategies by field operators. With the major categories of subsurface anomalies now identified, the development of automatic detection algorithms would allow a reliable identification of potentially problematic slabs or external defects affecting the RUP-FS structure, provided that sufficiently large datasets containing representative anomalies are available. A comprehensive understanding of subsurface defects will enable the anticipation and early prediction of surface distress development. Based on an improved understanding of wave–matter interaction physics, this manuscript proposes a non-automated subsurface classification approach grounded in realistic observation of the underlying phenomena. In future work, it may therefore be possible to identify relevant features to support automated detection and classification, and potentially enable data fusion with surface-based measurements.

The HERON project's multi-technique and multi-physics approach therefore integrates principles of targeted intervention and maintenance by identifying zones likely to develop

defects through automatic detection, aligning with the Forever Open Road (FOR) vision promoted by the Forum of European National Highway Research Laboratories (FEHRL) [54].

VI. Acknowledgments

The authors would like to thank the European Union's Horizon 2020 Research and Innovation Program (grant agreement No. 955356, Improved Robotic Platform to Perform Maintenance and Upgrading Roadworks: The HERON Approach), which made this study possible. The authors also express their gratitude to *Nantes Métropole* for its support in carrying out the experiments in the city center of Nantes.

References

[1] K. Khweir and D. Fordyce, "Influence of layer bonding on the prediction of pavement life", Proceeding of the Institution of Civil Engineers-Transport, 156, 2003, Issue TR2, pp.73-83, https://doi.org/10.1680/tran.2003.156.2.73

[2] F. de Larrard and J-M. Balay and T. Sedran and J-M. Masson and P. Duramé and R. Bélouard and P. Pommelet and D. Cante and G. Laurent and A. Leroux and J. Maribas and N. Vulcano-Greullet and G. Petit and G. Métais and L. Mancina and N. Sagnard and S. Bruny and C. Le Lez and L. Grin and J. Abdo and L. Brissaud, "Removable urban pavements: a new neighbour-friendly technology", XXIIIth World Road Congress, PIARC, Paris, France, 14(1), 2007.

[3] F. de Larrard and T. Sedran and J-M. Balay, "Removable urban pavements: an innovative sustainable technology", International Journal of Pavement Engineering, 14(1), ed Taylor & Francis, pp 1–11, 2013.

[4] A. Abdo and J-M. Balay and F. De Larrard and G. Laurent and A. Leroux and J-M. Masson and P. Pellevoisin and G. Petit and T. Sedran, "Chaussées urbaines démontables", Guide technique, edition du certu, ISBN 978-2-11-098199-1, décembre 2008, *in french*.

[5] I-Street Project (*in french*): https://librairie.ademe.fr/recherche-et-innovation/1619-i-street.html

[6] E. Gennesseaux and T. Sedran and J-M. Torrenti and M. Hardy, "Formulation of optimized excavatable cement treated materials using a new punching test apparatus", Mater Struct, 2018, 51: 56. https://doi.org/10.1617/s11527-018-1184-1, 2018

[7] T. Sedran and E. Gennesseaux and J. Waligora and P. Klein and M.L. Nguyen and J. Cesbron and J. Lelong and C. Ropert and L. Monnier, "Draining removable urban pavement: a modular solution for versatile urban streets", XXVIIe PIARC World Road Congress, Oct 2023, Prague (CZ), Czech Republic. ⟨hal-04416491⟩

[8] E. Gennesseaux and T. Sedran and and J. Waligora, "Excavatable and permeable cement sub-base for removable urban pavement (RUP)", 14th International Symposium on Concrete Roads, Jun 2023, Krakow, Poland, 2023.

[9] T. Sedran and E. Gennesseaux and J. Waligora and P. Klein and M.L. Nguyen and J. Cesbron and J. Lelong and C. Ropert and L. Monnier, "Development of a permeable removable urban pavement for a higher sustainability", Proceeding of 13th International Conference on Concrete Pavements, ISCP, Aug 2024, Minneapolis, United States. pp 299-311, ISBN 979-8-218-58882-3, ⟨hal-04723878⟩

[10] P. Klein and J. Cesbron and S. Bianchetti and E. Gennesseaux and T. Sedran and J. Waligora, "Porous top layer optimization of cement concrete slabs for tyre/road noise reduction", 51th Inter.noise conference, Glasgow, 21-24 august 2022

[11] P. Klein and J. Cesbron and E. Gennesseaux and T. Sedran, "Optimisation acoustique et évaluation in situ d'une chaussée urbaine démontable à surface fonctionnalisée", CFA 2025, 17e Congrès Français d'Acoustique, Société Français d'Acoustique (SFA), 2025, Paris, France, https://univ-eiffel.hal.science/hal-05210817v1, *in french*

[12] J. Cesbron and L. Rouy and V. Gary and J. Lelong and P. Klein and A. Le Bellec and E. Gennesseaux and T. Sedran, "On site acoustical characterization of a removable urban pavement with functionalized surface". 52nd Inter.noise conference, Aug 2023, Chiba greater Tokyo, Japan. pp.841-852, https://doi.org/10.3397/IN_2023_0135

[13] J. Blanc and P. Hornych and Z. Sotoodeh-Nia and C. Williams and L. Porot and S. Pouget, R. Boysen and J-P. Planche and D. Lo Presti and A. Jimenez and E. Chailleux, "Full-scale validation of bio-recycled asphalt mixtures for road pavement", Journal of Cleaner Production, vol. 227, pp 1068–78, 2019, https://doi.org/10.1016/j.jclepro.2019.04.273

[14] D. Arya and H. Maeda and S.K. Ghosh and D. Toshniwal and A. Mraz and T. Kashiyama and Y. Sekimoto, "Deep learning-based road damage detection and classification for multiple countries", Automation in Construction, 132, 2021, 103935, https://doi.org/10.1016/j.autcon.2021.103935

[15] I. Katsamenis and E. Protopapadakis and A. Doulamis and N. Doulamis and A. Voulodimos, "Pixel-level corrosion detection on metal constructions by fusion of deep learning semantic and contour segmentation", In International Symposium on Visual Computing. Springer, 160–169, 2020.

[16] J. Liu and H. Luo and H. Liu, "Deep learning-based data analytics for safety in construction", Automation in Construction, vol. 140, 2022, 104302.

[17] The HERON Project: https://www.heron-h2020.eu/

[18] Improved Robotic Platform to perform Maintenance and Upgrading Roadworks: The HERON Approach, 2025, https://cordis.europa.eu/project/id/955356/reporting, DOI: 10.3030/955356

[19] S. Stavridis and L. Droukas and Z. Doulgeri and D. Papageorgiou and F. Dimeas and A. Soriano and S. Molina and S. Deiri and M. Hutchinson J. Pulido-Fentanes and I. Hroob and R. Polvara and M. Hanheide and G. Cielniak and N. Samarinas and D. Kateris and D. Bochtis and G. Peleka and S. Papadam and D. Triantafyllou and A. Papadimitriou and C. Papadopoulos and I. Mariolis and D. Giakoumis and D. Tzovaras, "Robotic Grape Inspection and Selective Harvesting in Vineyards: A Multisensory Robotic System With Advanced Cognitive Capabilities", IEEE Robotics & Automation Magazine, PP. 2-14, 2024, 10.1109/MRA.2024.3487324.

[20] I. Katsamenis and M. Bimpas and E. Protopapadakis and C. Zafeiropoulos and D. Kalogeras and A. Doulamis and N. Doulamis and C. Martín-Portugués Montoliu and Y. Handanos and F. Schmidt and L. Ott and M. Cantero and R. Lopez, "Robotic maintenance of road infrastructures: The heron project", 2022, In Proceedings of the 15th International Conference on PErvasive Technologies Related to Assistive Environments, 628–635, https://doi.org/10.1145/3529190.3534746

[21] I. Katsamenis and N. Doulamis and A. Doulamis and E. Protopapadakis and A. Voulodimos, "Simultaneous Precise Localization and Classification of metal rust defects for robotic-driven maintenance and prefabrication using residual attention U-Net", 2022, Automation in Construction, 137, 104182, https://doi.org/10.1016/j.autcon.2022.104182

[22] Q. Mei and M. Gül, "A cost effective solution for pavement crack inspection using cameras and deep neural networks", Construction and Building Materials, 256, 2020, 119397, https://doi.org/10.1016/j.conbuildmat.2020.119397

[23] N. Bahrani, F. Schmidt, and N. Hautiere, "A comprehensive study & outlook on integrated structural health monitoring and C-intelligent transport system for pavement condition assessment", Transportation Research Procedia, 72, 2023, 4231–4238, https://doi.org/10.1016/j.trpro.2023.11.350

[24] L.A. Silva and H. Sanchez San Blas and D. Peral García and A. Sales Mendes and G. Villarubia González, "An architectural multiagent system for a pavement monitoring system with pothole recognition in UAV images", Sensors, 20, 21, 2020, 6205, https://doi.org/10.3390/s20216205

[25] I. Colomina and P. Molina, "Unmanned aerial systems for photogrammetry and remote sensing: A review", ISPRS Journal of photogrammetry and remote sensing, 92, 2014, 79–97, https://doi.org/10.1016/j.isprsjprs.2014.02.013

[26] I. Katsamenis and N. Bakalos and E. Protopapadakis and E.E. Karolou and G. Kopsiaftis and A. Voulodimos, "Real time road defect monitoring from UAV visual data sources", in PETRA '23: Proceedings of the 16th International Conference on Pervasive Technologies Related to Assistive Environment, pp 603-609, 2023, https://doi.org/10.1145/3594806.3596561

[27] Z. Jiang and L. Zhao and S. Li and Y. Jia, "Real-time object detection method based on improved YOLOv4-tiny", ArXiv, abs/2011.04244, 2020, https://doi.org/10.48550/arXiv.2011.04244

[28] A. Bochkovskiy and C-Y. Wang and H-Y. Mark Liao, "YOLOv4: Optimal Speed and Accuracy of Object Detection", 2020, https://doi.org/10.48550/arXiv.2004.10934

[29] J. Redmon and S. Divvala and R. Girshick and A. Farhadi, "You only look once: Unified, real-time object detection", In Proceedings of the IEEE conference on computer vision and pattern recognition, 779–788, 2016.

[30] I. Katsamenis and A. Davradou and E. Eirini Karolou and E. Protopapadakis and A. Doulamis and N. Doulamis and D. Kalogeras. , "Evaluating YOLO transferability limitation for road infrastructures mon-

itoring", In Novel & Intelligent Digital Systems Conferences, Springer, 349–358, 2022

[31] G. Andreoli and F. Schmidt and A. Ihamouten and A. Cothenet and T. Dezert and M.L. Nguyen and T. Sedran, "Subsurface characterization of Removable Urban Pavements (RUP) using Ground Penetrating Radar (GPR)", Journal of Physics: Conference Series, 2887, 2024, https://doi.org/10.1088/1742-6596/2887/1/012071

[32] I. Katsamenis and G. Andreoli and M. Skamantzari and N. Bakalos and F. Schmidt and T. Sedran and N. Doulamis and E. Protopapadakis and D. Kalogeras, "UAV-based Localization of Removable Urban Pavement Elements Through Deep Object Detection Methods", in PETRA '24: Proceedings of the 17th International Conference on Pervasive Technologies Related to Assistive Environment, pp 440-448, 2024, https://doi.org/10.1145/3652037.3663934

[33] DJI official site: https://www.dji.com/fr

[34] F. Seraj and B. Jan Van Der Zwaag and A. Dilo and T. Luarasi and P. Havinga, "RoADS: A road pavement monitoring system for anomaly detection using smart phones", In International Workshop on Modeling Social Media, Springer, 128–146.

[35] Kontūr official site: https://www.kontur.tech/

[36] L. Perez and J. Wang, "The Effectiveness of Data Augmentation in Image Classification using Deep Learning", Technical report, 2017, 10.48550/arXiv.1712.04621.

[37] A. Mikołajczyk-Bareła and M. Grochowski, "Data augmentation for improving deep learning in image classification problem", International Interdisciplinary PhD Workshop (IIPhDW), 117-122, 2018, DOI: 10.1109/IIPHDW.2018.8388338.

[38] A. Kwasigroch and A. Mikołajczyk and M. Grochowski, "Deep convolutional neural networks as a decision support tool in medical problems–malignant melanoma case study", Trends in Advanced Intelligent Control, Optimization and Automation, Advances in Intelligent Systems and Computing, In: Mitkowski W., Kacprzyk J., Oprzędkiewicz K., Skruch P. (eds), vol 577. Springer, Cham, pp. 848–856, 2017.

[39] A. Kwasigroch and A. Mikołajczyk and M. Grochowski, "Deep neural networks approach to skin lesions classification — A comparative analysis", 22nd International Conference on Methods and Models in Automation and Robotics (MMAR), Miedzyzdroje, 2017, pp. 1069-1074, 2017.

[40] M. Wąsowicz and M. Grochowski and M. Kulka and A. Mikołajczyk and M. Ficek and K. Karpieńko and M. Ćićkiewicz, "Computed aided system for separation and classification of the abnormal erythrocytes in human blood", in Biophotonics—Riga 2017, vol. 10592, p. 105920A, 2017.

[41] A. Galdran and A. Alvarez-Gila and M.I. Meyer and C.L. Saratxaga and T. Araújo and E. Garrote and G. Aresta and P. Costa and A.M. Mendonça and A. Campilho, "Data-Driven Color Augmentation Techniques for Deep Skin Image Analysis", 2017, https://doi.org/10.48550/arXiv.1703.03702

[42] G. Yao and Y. Sun and M. Wong and X. Lv, "A Real-Time Detection Method for Concrete Surface Cracks Based on Improved YOLOv4", Symmetry, vol. 13, 1716, 2021, DOI: 10.3390/sym13091716

[43] J-A. Sarmiento, "Pavement Distress Detection and Segmentation using YOLOv4 and DeepLabv3 on Pavements in the Philippines", 2021, https://doi.org/10.48550/arXiv.2103.06467.

[44] C. Guo and X-L. Lv and Y. Zhang and M-L. Zhang, "Improved YOLOv4-tiny network for real-time electronic component detection", Sci Rep 11, 22744, 2021, https://doi.org/10.1038/s41598-021-02225-y

[45] W. Feng and Y. Zhu and J. Zheng and H. Wang and X. Chen, "Embedded YOLO: A Real-Time Object Detector for Small Intelligent Trajectory Cars", Mathematical Problems in Engineering, Hindawi, vol. 2021, pages 1-11, 2021, https://doi.org/10.1155/2021/6555513

[46] Y. Jiang and W. Li and J. Zhang and F. Li and Z. Wu, "YOLOv4-dense: A smaller and faster YOLOv4 for real-time edge-device based object detection in traffic scene", IET Image Process, 17, 570580, 2023, https://doi.org/10.1049/ipr2.12656

[47] A. Bakar and K. Li and H. Liu and Z. Xu and M. Alessandrini and D. Wen, "Multi-Objective Optimization of Low Reynolds Number Airfoil Using Convolutional Neural Network and Non-Dominated Sorting Genetic Algorithm", Aerospace, vol. 9, 35, 2022, DOI: 10.3390/aerospace9010035

[48] D-J. Shin and J-J. Kim, "A Deep Learning Framework Performance Evaluation to Use YOLO in Nvidia Jetson Platform", MDPI, Data Analysis and Artificial Intelligence for IoT, 2022, https://doi.org/10.3390/app12083734

[49] C-Y. Wang and A. Bochkovskiy and H-Y. M. Liao, "Scaled-YOLOv4: Scaling Cross Stage Partial Network", Conference on Computer Vision and Pattern Recognition (CVPR), 2021, https://doi.org/10.1109/CVPR46437.2021.01283

[50] J. Foster and L. Ott and J. Nieto and N. Lawrance and R. Siegwart, (2023). "Automatic Extension of a Symbolic Mobile Manipulation Skill Set", Robotics and Autonomous Systems, 2023, https://doi.org/10.1016/j.robot.2023.104428

[51] HERON Deliverables, 2021,
https://www.heron-h2020.eu/?page_id=1115

[52] W.A. Schneider, "Integral Formulation for Migration in Two and Three Dimensions", Journal of Geophysics, 43(1), 49–76, 1978, https://doi.org/10.1190/1.1440828

[53] K. Meyer and E. Erdogmus and G. Morcous and M. Naughtin, "Use of Ground Penetrating Radar for Accurate Concrete Thickness Measurements", Proceedings of the AEI 2008 Conference - AEI 2008: Building Integration Solutions, 328, 1-10, 2008, https://doi.org/10.1061/41002(328)67

[54] Forever Open Road: https://foreveropenroad.fehrl.org/
